# The fetal ovary exhibits temporal sensitivity to a ‘real-life’ mixture of environmental chemicals

**DOI:** 10.1038/srep22279

**Published:** 2016-03-02

**Authors:** Richard G. Lea, Maria R. Amezaga, Benoit Loup, Béatrice Mandon-Pépin, Agnes Stefansdottir, Panagiotis Filis, Carol Kyle, Zulin Zhang, Ceri Allen, Laura Purdie, Luc Jouneau, Corinne Cotinot, Stewart M. Rhind, Kevin D. Sinclair, Paul A. Fowler

**Affiliations:** 1 Schools of Veterinary Medicine and Biosciences, University of Nottingham, Leicestershire, LE12 5RD, UK; 2 Institute of Medical Sciences, School of Medicine, Medical Sciences & Nutrition, University of Aberdeen, Foresterhill, Aberdeen, AB25 2ZD, UK; 3 UMR BDR, INRA, Université Paris Saclay, 78350, Jouy-en-Josas, France; 4 The James Hutton Institute, Craigiebuckler, Aberdeen, AB15 8QH, UK

## Abstract

The development of fetal ovarian follicles is a critical determinant of adult female reproductive competence. Prolonged exposure to environmental chemicals (ECs) can perturb this process with detrimental consequences for offspring. Here we report on the exposure of pregnant ewes to an environmental mixture of ECs derived from pastures fertilized with sewage sludge (biosolids): a common global agricultural practice. Exposure of pregnant ewes to ECs over 80 day periods during early, mid or late gestation reduced the proportion of healthy early stage fetal follicles comprising the ovarian reserve. Mid and late gestation EC exposures had the most marked effects, disturbing maternal and fetal liver chemical profiles, masculinising fetal anogenital distance and greatly increasing the number of altered fetal ovarian genes and proteins. In conclusion, differential temporal sensitivity of the fetus and its ovaries to EC mixtures has implications for adult ovarian function following adverse exposures during pregnancy.

The deleterious effects of exposure to environmental chemicals (ECs), including endocrine disrupting compounds (EDCs), on female reproductive function have been demonstrated in diverse species^1^,^2^, especially at immature life stages^3^,^4^. However, most mechanistic studies have been performed in rodents using single chemicals [e.g.^5^], but in these species ovarian follicle assembly begins two days before birth and oocytes are only enclosed in follicles during the first few days after birth, unlike the human^6^. Therefore, we previously subjected pregnant ewes to a complex mixture of ECs for a prolonged period by grazing pastures fertilized with sewage sludge (biosolids); a common agricultural practice. This valuable fertilizer contains readily detectable levels of EDCs^7^,^8^,^9^,^10^ which, through release into the environment, represents animal and human exposure. Indeed, the very complexity of the EC mixture, which would be problematic to reproduce artificially, provides a means of investigating the effects of real-life maternal exposure on the fetus. We have reported that prolonged use of sewage sludge fertilizer increases the soil content of many ECs/EDCs^11^. Pertinently, approximately 8 million dry metric tonnes of sludge are produced in the USA and EU per annum. This translates to a sludge density (dry metric tonne/annum/km^2^) of 1.6 in the USA and 4.6 in the EU^7^. Whilst ECs in pregnant ewe tissues are minimally affected by sewage sludge exposure^8^,^9^, concentrations of ECs in maternal and fetal livers are generally different. Some ECs appear to be present at higher levels in the fetus (e.g. PCB 101) whilst others (e.g. DEHP) show accumulation in both mother and fetus^9^. Despite these observations, fetuses from ewes following prolonged exposure exhibit disturbed ovarian^10^,^11^, testicular^12^, thyroidal^13^ and hypothalamo-pituitary axis^14^,^15^ development. Adult male offspring exposed both pre- and post-natally, exhibited perturbed testicular morphology^16^ and feminized behavior^17^.

Ovarian development in precocial mammals, including humans, extends across much of gestation. Within the context of environmental perturbation, identification of stages of ovarian development most influenced by ECs requires rigorously controlled exposure at different stages of gestation and associated measurements of: (a) tissue chemical burdens, (b) indices of fetal, particularly ovarian development, and (c) patterns of gene and protein expression. We therefore exposed pregnant ewes, at different stages of gestation, to pastures fertilized with sewage sludge. Exposure occurred during three overlapping 80-day periods depicted in Fig. 1. These encompassed early (0–80 dpc), mid (30–110 dpc), and late (60–140 dpc) gestation. Exposures were compared to controls (inorganic fertilizer) and to continuous sewage sludge exposure (0–140 dpc). During days 0–80, the gonads differentiate (25 dpc), primordial germ cells migrate to and proliferate in the gonads (25–45 dpc), meiotic prophase I occurs (55–60 dpc) and primordial follicles form (75–80 dpc)^18^,^19^. Fetal hypothalamo-pituitary function develops after 60 dpc^20^, and by 140 days the first antral follicles are present^21^, with birth at 145–150 dpc. Using this model we establish the gestational periods during which the fetal ovary is most sensitive to perturbation by a complex, low dose, mixture of ECs.

## Results

### Effects of sludge exposure on 31 ECs in fetal and maternal liver

Soil concentrations of ECs were increased by sewage sludge application, especially polycyclic aromatic hydrocarbons (PAHs): ∑16 PAHs increased from 1812 to 3162 μg/kg soil. In addition, ∑7 polychlorinated bisphenyl (PCB) congeners rose from 0.11 to 0.14 μg/kg soil whereas ∑7 polybrominated diphenyl ethers (PBDEs) remained constant at 0.60 μg/kg soil. Di(2-ethylhexyl)phthalate (DEHP) also increased from 60 to 80 μg/kg soil.

In maternal livers, 10 of the 15 significant alterations in chemical concentrations were manifest by an increased chemical burden following exposure to sludge treated pastures (Supplementary Fig. S1). Significant differences in some PAH, PBDE and PCB congeners only occurred in the 30–110T and 60–140T groups. DEHP was increased in all 3 transient exposure groups (0–80T, 30–110T, 60–140T). Fetal liver EC concentrations at Day 140 were highly variable and the only significant differences from controls (0–140C) were in the 30–110T and 60–140T groups (Supplementary Fig. S2), with significant increases only in the 30–110T group (PBDE100, PCB52). In contrast, benzo(a)anthracene and specific PBDE (28,47) and PCB (28,101,118,138,153,180) congeners were decreased in one or both of the mid and late exposure groups. Consistent with previous studies^8^,^22^,^23^, measured fetal and maternal liver EC concentrations could not be equated with concentrations in soil e.g. PAH chemicals (Supplementary Table S1).

### Effects of sludge exposure on maternal body condition score and fetal urogenital tract and endocrinology

Exposure to sewage sludge had no effect on maternal BCS (0–140C: 2.29 ± 0.04, 0–140T: 2.21 ± 0.04, 0–80T: 2.21 ± 0.04, 30–110T: 2.19 ± 0.15, 60–140T: 2.09 ± 0.05). Day 140 female fetal mass in all but one (i.e. 0–140T) exposure group was lower (P < 0.001) than controls (Table 1). Normalized (for fetal mass) fetal anogenital distances (AGD) were greater (P < 0.05) in fetuses of the 30–110T and 60–140T exposure groups relative to 0–140C fetuses. Normalized fetal liver mass was reduced (P < 0.05) in the 0–140T group but increased (P < 0.05) in the 60–140T group. Normalized fetal thyroid and uterine mass in the 60–140T group was increased (P < 0.05) and reduced respectively relative to controls. Fetal hormones were altered (P < 0.05) in the 0–80T group (testosterone increased) and in the 60–140T group (free T3 and T4 were reduced and increased respectively).

### Effects of sludge exposure on ovarian development

Normalized fetal-ovarian mass was not affected in any sludge exposure group (Table 1). Overall follicular density (number/mm^2^) was not affected by exposure [All follicles: 0–140C: 17.57 ± 7.10, 0–140T: 18.42 ± 3.34, 0–80T: 18.67 ± 4.32, 30–110T: 28.03 ± 3.75, 60–140T: 34.25 ± 6.77] and similarly, the percentage of all follicles in each follicle type in the day 140 ovaries did not alter across groups [Type 0: 3.3 ± 0.7%, Type 1: 20.8 ± 2.1%, Type 1a: 63.0 ± 1.3%, Type 2: 14.6 ± 2.3%, Types 3–5: 1.7 ± 0.4%]. However, the proportion of healthy post-primordial transitory follicles (i.e. Type 1a: Fig. 2a.ii), representing the largest class of Day 140 fetal-ovarian follicle, was reduced (P < 0.05) in all exposure groups (Fig. 2b, Table 2). This corresponded with a separately-counted increase in the proportion of atretic Type 1a follicles (Fig. 2a.iv,b) and an increased proportion of follicles with intense nuclear staining (Fig. 2a.v) specific to the 0–80T and 30–110T groups (Table 2). In contrast, earlier stage type 1 follicles (Fig. 2a.i,a.ii) exhibited an increased proportion of healthy follicles in the 60–140T group and an apparent decrease in atretic follicles in all exposure groups (Table 2). At the next developmental stage (Type 2; Fig. 2a.iii), the exposure effect was still evident with a significant increase in numbers of Type 2 follicles in the 60–140T group (Table 2). However, the decline in the proportion of healthy follicles reached significance in only the 30–110T group which also exhibited an increased proportion of follicles with intense nuclear staining.

### Effects of sludge exposure on the fetal ovarian transcriptome

Gene array analysis revealed that 256 transcripts (3.4% of 7,500) were differentially expressed (P < 0.05 after Benjamini-Hochberg multiple testing correction (FDR at 5%) and absolute fold-change (aFC) >1.5) between controls and exposed fetal ovaries. Of these, 33 were differentially expressed in continuously exposed fetuses (0–140T) but only 4 were differentially expressed in the 0–80T group (Fig. 3a and Supplementary Table S2A). In contrast, 99 and 120 transcripts were differentially expressed in the 30–110T and 60–140T groups respectively. These exposure periods embrace oocyte prophase I meiosis (50–75 dpc in sheep) and follicle formation (75–140 dpc in sheep). In all exposure conditions, 76% of transcripts were down-regulated (196/256) vs 23% (60/256) upregulated (Fig. 3a). Strikingly, few differentially expressed transcripts were common to two or more periods of exposure; that is the majority were specific to single exposure periods (Fig. 3b). For example, only 22 transcripts were altered in any two specific periods and only *IGHA2* was altered in all four (Fig. 3b, Supplementary Table S2B). Differentially expressed transcripts were associated with cellular growth and differentiation, cell cycle regulation, cell death, cellular development, and cell movement (IPA analysis; Fig. 3c). These cellular functions are active during follicle formation when pre-granulosa cells proliferate and move to surround oocytes. These functions were particularly affected during the 60–140 dpc period (Fig. 3c, Supplementary Table S2A). The ERK/MAPK and PI3K/AKT signaling, growth hormone signaling and actin cytoskeleton signaling pathways were the most affected by sewage sludge exposure (Fig. 3d). These are all involved in dialogue between germ cells and somatic cells during early folliculogenesis.

Fourteen of the differentially expressed genes are involved in DNA methylation, with 10 identified as methyl transferases, 3 as demethylases and 1 as a histone deacetylase subunit (Supplementary Table S3). Significant differences were most evident in the late (60–140T) gestation exposure groups. Six of nine differentially expressed genes detected by microarray showed identical directional changes (up or down regulation) by qPCR, four of which were significant (Supplementary Table S4).

### Effects of sewage sludge exposure on the fetal ovarian proteome

Proteomic analyses revealed 64/776 analyzed protein spots (Fig. 4a), normalized for volume, that were altered (P < 0.05) relative to controls. Significantly, 30–110T and 60–140T groups clustered separately from 0–140 C, 0–140T and 0–80T (Fig. 4b). In contrast to transcript expression, 83% of these protein spots were up-regulated and, relative to controls, the 30–110T and 60–140T groups displayed the greatest number of differentially expressed spots (Fig. 4c). Methylenetetrahyrdofolate dehydrogenase 1 (Fig. 4d) and Serotransferrin (Fig. 4e) represent examples of up- and down-regulation respectively during the 30–110 and 60–140 periods of exposure (see also Supplementary Table S5).

Only two protein spots were altered in all treatment groups and these were identified as Lamin A/C and Lamin M/C (up-regulated) (Fig. 4f, Supplementary Table S5). We elected to verify quantification for these two proteins by Western blot which confirmed that their up-regulation was attributable to the 65 kDa *LMNA* (LAMIN C) product and that this was restricted to the 0–140T, 0–80T and 30–110T groups (Fig. 5a,b, Supplementary Fig. S3). In contrast, the 75 kDa product (LAMIN A) was unaffected. Immunocytochemistry revealed that LAMIN A/C was noticeable in most cells but was more condensed in granulosa cells than in the oocyte or stromal cells (Fig. 5c).

Pathway analysis of the most consistently differentially expressed proteins across all treatments revealed two networks primarily concerned with tissue defense and metabolism (Supplementary Table S5). Proteins selected from both networks were expressed predominantly in the oocyte cytoplasm (e.g. UCHL1, PDIA3, GSTM3, HSPA9 and BLVRA) (Supplementary Fig. S4). In contrast, COPS4 was detected in the nucleus of granulosa cells and the cytoplasm of oocytes, whereas GSN was detected in the nucleus of both cell types.

## Discussion

The findings from the current study are significant for the following reasons. They identify stages of female-fetal gonad development in sheep that are particularly sensitive to environmental-chemical exposure, which in our case arose when ewes grazed sewage-sludge treated pastures. Whilst EC exposure reduced the proportion of healthy primordial/transitory follicles regardless of developmental stage, exposure during the latter stages of gestation led to the most divergent concentrations of fetal liver chemicals and, significantly, differentially expressed transcripts and proteins essential for normal ovarian development. Given that AGD was also increased in these fetuses, collectively, these observations present the tantalizing prospect that EC exposure from mid pregnancy could have a more profound and permanent effect on ovarian function that extends beyond our initial observations on fetal follicular development to influence fecundity more generally during adulthood. To date these long-term consequences have not been explored.

Previous studies by our group assessed the effects of sludge exposure on fetal ovary development continuously up to Day 110 of gestation (i.e. for 0.75 of gestation)^10^,^11^. Consistent with our current observations, these two studies observed a significant reduction in the proportion of Type 1a follicles. Thus we now provide compelling evidence from three independent studies that exposure to ECs from sewage sludge profoundly alters early follicle development in the fetal ovary. The present study sought to determine if there was a key EC sensitive stage of fetal ovarian development, as has been reported for the testis in rodents^24^. However, it would appear that EC exposure during any or all of the stages of gestation studied affects fetal folliculogenesis in a phenotypically similar manner (Fig. 2). This could reflect the common period of exposure (i.e. Day 60 to 80; coincident with primordial follicle formation) for all treatment groups. In contrast, the greatest differences in both transcript and protein expression in the fetal ovary were observed when EC exposure occurred from around mid-gestation (Figs 3 and 4), coinciding with processes of oocyte meiosis through to primary follicle differentiation, leading onto antral-follicle formation^25^.

The increase in AGD observed in 30–110T and 60–140T groups, together with the observed increase in liver and decrease in uterine mass for the 60–140T (Table 1) may be indicative of androgenization^26^,^27^,^28^. In support of this, pregnant ewes treated with testosterone propionate (TP) within the same time frame (30–90 days of gestation) generated female offspring with increased AGD and a depleted pool of primordial follicles^29^,^30^. In keeping with the current study, Smith *et al.*^30^ also reported an increase in the number of type 2 follicles at day 140. Collectively these observations implicate a role for androgens in the dysregulation of follicle development. However, from the profile of ECs accumulated in the fetal liver (Supplementary Fig. S2) it is difficult to predict whether a more androgenic environment had been created in our treatment groups. In general, fetal tissue concentrations of most PDBE and PCB congeners decreased for these groups; the exceptions being congeners PBDE100 and PCB52, which were markedly increased. These two classes of EC are generally considered to have pro-estrogenic or anti-androgenic actions^31^. However, the effect of any overall mixture of chemicals depends on their precise combination^32^, so making it difficult to predict the effects of individual ECs in this study.

As previously stated, the transient exposure of pregnant ewes to sewage sludge treated pastures from 30–110 and 60–140 days led to a greater number of differentially expressed fetal-ovarian transcripts and proteins than observed following continuous exposure. In a previous cross-over study, we reported that switching dams from control to sewage-sludge treated pastures at conception, and *vice versa*, also leads to a greater number of altered ovarian transcripts at day 110 than observed with continuous exposure^11^. In both studies, this may reflect altered maternal and/or fetal metabolism during pregnancy secondary to changes in pharmacokinetic parameters. One possible mechanism is that exposure induced changes in maternal liver, placental and/or fetal liver metabolising enzyme expression or activity early in gestation may attenuate the effects of the same chemicals on the late gestation fetus through adaptive processes. In support of this premise, some human fetal liver DMEs are expressed at their highest levels during early pregnancy^33^ and we have shown in the human that already in the second trimester the fetal liver responds to challenges, as evinced in our other “real-life” exposure model, maternal cigarette smoking^34^,^35^,^36^,^37^. In the current study, pathway analysis of differentially expressed fetal ovarian protein spots highlighted drug metabolism as one of the top functions (Supplementary Table S6). Furthermore, a greater number of these proteins were altered in the 30–110T and 60–140T groups than in the 0–80T group, or in continuously exposed animals. Transcript analyses also identified differentially expressed DMEs, exemplified by the cytochrome P450 (CYP) enzyme genes^38^. Although in the current model, DME expression in liver (fetal and/or maternal) or the placenta was not investigated, our observations on fetal ovarian DMEs, suggest that the differential expression of DMEs may account for some of the differential effects on fetal outcomes between exposure groups. It is possible therefore that continuous exposure to ECs contained in sewage sludge may have altered DME expression differently to that observed in the transient exposure groups. Differential DME expression may also account for the differences in EC load between animals exposed for different but overlapping periods. Consistent with previous studies^8^,^22^,^23^, tissue EC variability may also reflect the complex physiological and “between chemical” interactions that occur during maternal ingestion and digestion and the catabolic/anabolic status of the individual animal, particularly given the lipophilic property of many ECs.

The 12 signaling pathways significantly altered by sewage sludge exposure (Fig. 3d) were most perturbed in the 60–140T group. The PI3K/AKT pathway has been identified as a major canonical pathway in quiescent human primordial follicles whereas ERK/MAPK becomes enriched in mature metaphase II oocytes^39^. Both pathways are affected in the 60–140T fetal ovaries and the dysregulation of the PTEN/PI3K pathway has been linked to ovarian failure and infertility^40^,^41^. Extending beyond the ovary, PCB-perturbed thyroid function has been linked to the dysregulation of the insulin/PI3K pathway^42^,^43^. Perturbed thyroid development and altered T3 to T4 ratio reported in the current study (Table 1) may therefore operate through a similar mechanism.

Since all fetal ovaries were collected at Day 140, the profile of transcripts and proteins expressed at this stage reflects a snapshot of mechanistic changes that, by virtue of developmentally regulated gene expression, may not relate temporally to the profile of Day 140 follicles. Furthermore, we recognize that the use of whole fetal ovarian tissue rather than isolated ovarian cell types provides an interpretational limitation on the data generated. Nevertheless, some useful insights into potential mechanisms of impaired ovarian development can be inferred. For example, transcript analyses highlighted cellular processes critical for follicle assembly and development (Fig. 3c) and IPA analysis of all transcripts and proteins altered in the 60–140T group predicted a decreased activation state of these processes (Supplementary Table S7). At a functional level, IPA network analyses highlighted roles additional to drug metabolism including free radical scavenging, metabolic disease and amino acid metabolism (Supplementary Tables S5 and S6).

The trend for down-regulation of differentially expressed genes in the 30–110T and 60–140T groups is consistent with ECs causing a cascade of events mediated by epigenetic mechanisms leading to the silencing of gene expression (reviewed in^44^). Specific ECs are known to alter epigenetic states including the susceptibility of histone methylation marks to endocrine disruption^45^. In the current study, 14 genes coding for histone methylation (Supplementary Table S3) were differentially expressed while fetal ovarian methylenetetrahydrofolate dehydrogenase (NADP + dependent) 1 (MTHFD1) protein, a folate-metabolizing enzyme necessary for cellular methylation, was increased specifically in the 30–110 and 60–140T groups (Fig. 4d, Supplementary Table S5). The deregulation of MTHFD1 protein expression could contribute to altered DNA/histone methylation in exposed ovaries and lead to perturbed gene expression. To investigate this further, IPA analysis, based entirely on this protein, and genes encoding epigenetic regulators identified in the current study, predicted a decrease in RNA/DNA expression/transcription, an increase in cell proliferation and a decrease in cell death (Supplementary Fig. S5): features generally consistent with our experimental observations.

In conclusion, this study demonstrates that the ovine fetal ovary exhibits differential and temporal sensitivity to a ‘cocktail’ of chemicals present in sewage sludge treated pastures. Although transitory type 1a pre-primary follicle development is sensitive to exposure across all developmental stages, exposure during mid and late gestation induced the most divergent concentrations of liver chemicals coincidentally with the largest number of altered ovarian transcripts and proteins. Cellular functions and signaling pathways consistent with follicle activation and development to the primary follicle stage were perturbed. Transcripts for key epigenetic regulators were also altered. Indeed given the overall down-regulation in transcript expression observed, these findings are indicative of global epigenetic dysregulation, although this was not determined in the current study. These data, however, highlight risks associated with the standard global agricultural practice of grazing livestock on pastures fertilized with treated human and industrial effluents. Since low-level chemical exposure poses a threat to human reproductive development, the consumption of products from animals grazing such pastures represents a substantial environmental concern.

## Methods

### Animals, treatment groups and tissue collection

All experiments were performed in accordance with relevant guidelines and regulations and all experimental protocols were approved by the James Hutton Institute’s Local Ethical Committee and fully licensed by the United Kingdom’s Animals Scientific Procedures Act 1986 under Project license authority (60/3356).

**Figure 1:**
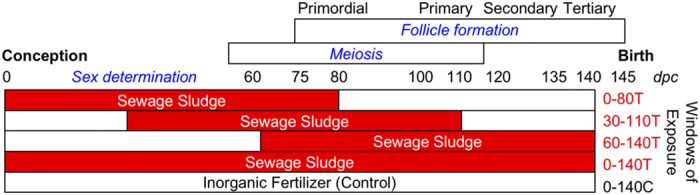
Experimental paradigm to investigate the effects of sludge exposure on the fetal ovary limited to 80 day periods during early, mid or late gestation. Pregnant ewes were exposed to sewage-sludge fertilized (T) pastures from: days 0 to 140 (0–140T, continuous exposure), days 0 to 80 (0–80T, early exposure), days 30 to 110 (30–110T, mid exposure) and days 60 to 140 (60–140T, late exposure). Control group pregnant ewes (C) were maintained on pastures fertilized with inorganic fertilizer from days 0 to 140 of gestation (0–140C).

**Figure 2:**
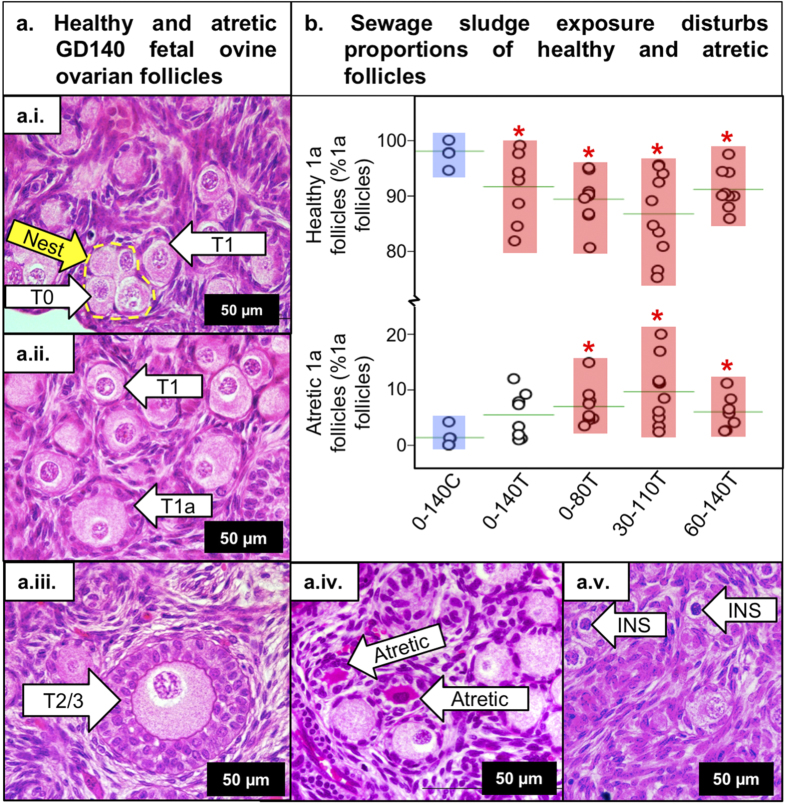
Follicle development in the Day 140 fetal ovary following continuous (0–140T) or transitory (0–80T, 30–110T and 60–140T) maternal exposure to sludge treated pastures. (**a**) Healthy follicle stages in developmental order (a.i to a.iii) include Type 1 (primordial), Type 1a (transitory/activated), Type 2 (primary), and Type 3 (small-preantral). Atretic follicles (a.iv: cytoplasm shrinkage from granulosa cells, dark coloration) and those with intense nuclear staining (INS: a.v). Scale bar = 50 μm. (**b**) The proportion of healthy Type 1a follicles was reduced (P < 0.05) in all exposure groups. Separately-counted atretic Type 1a follicles were increased (P < 0.05) in all transitory exposure groups. Developmental types: type 0 = isolated or naked oocyte (without associated granulosa cells (GC). Follicle types (Lundy *et al.*^46^): type 1 = primordial follicle (oocyte with flattened GC; type 1a = Transitory/activated follicle (a mixture of flattened and ≥1 cuboidal GC; type 2 = primary follicle (1 to <2 complete layers of cuboidal GC; type 3 = small pre-antral follicle (2 to <4 complete layers of cuboidal GC); type 4 = large pre-antral follicle (4 to <6 layers of cuboidal GC); type 5 = small antral follicle (>5 layers cuboidal GC).

**Figure 3:**
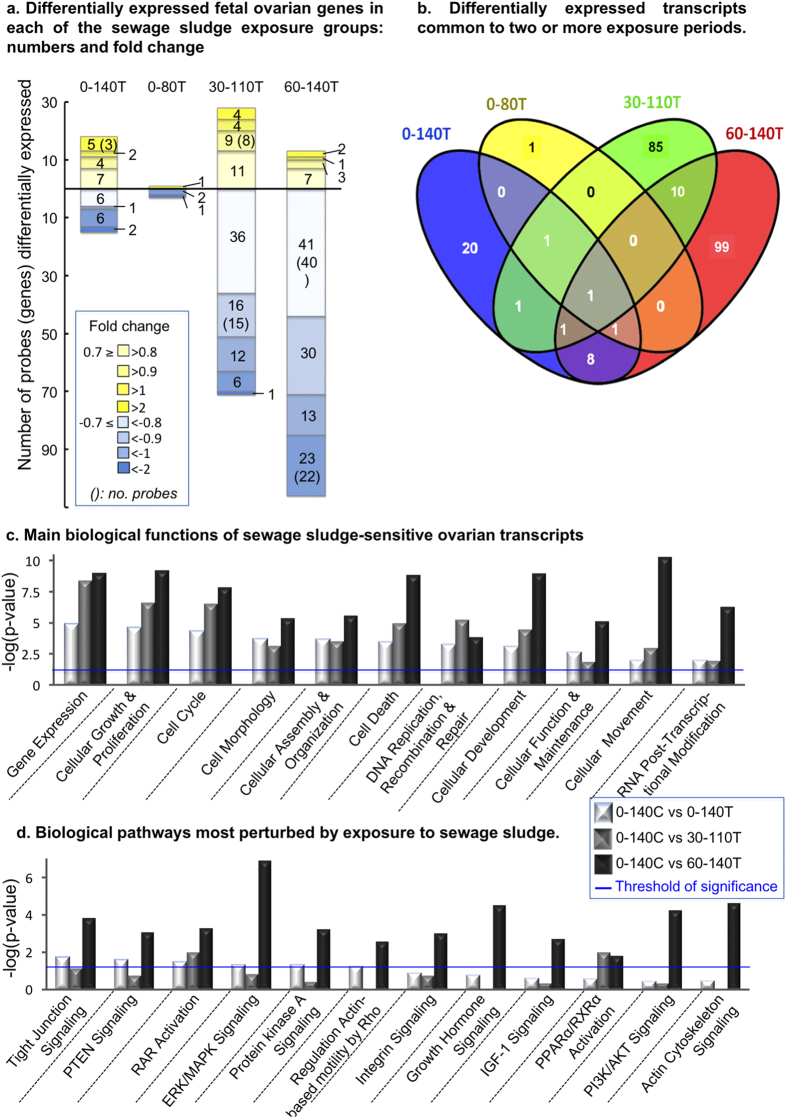
Analysis of differentially expressed (relative to Control) fetal ovarian genes following maternal sludge-exposure. (**a**) More genes were altered following exposure in the 30–110T and 60–140T groups than in the 0–140T and 0–80T groups. Only probes that met an FDR of 5% and a threshold of ±0.7 on the log2 transformed fold change (LogFC) are displayed. For each exposure, probes were divided into categories according to their positive (yellow) or negative (blue) fold change. (**b**) Venn diagram depicting the number of differentially expressed transcripts by exposure group. Few differentially expressed transcripts were common to two or more periods of exposure. (**c**) Functional analysis of differentially expressed transcripts (FDR ≤5%; LogFC threshold ± 0.2) representing a number of specific biological pathways (**d**) was performed with Ingenuity Pathway Analysis (IPA) software. The few transcripts that were altered in the 0–80T group were excluded. Significance was computed by Fisher’s exact test and the horizontal blue line corresponds to the P-value threshold (P ≤ 0.05).

**Figure 4:**
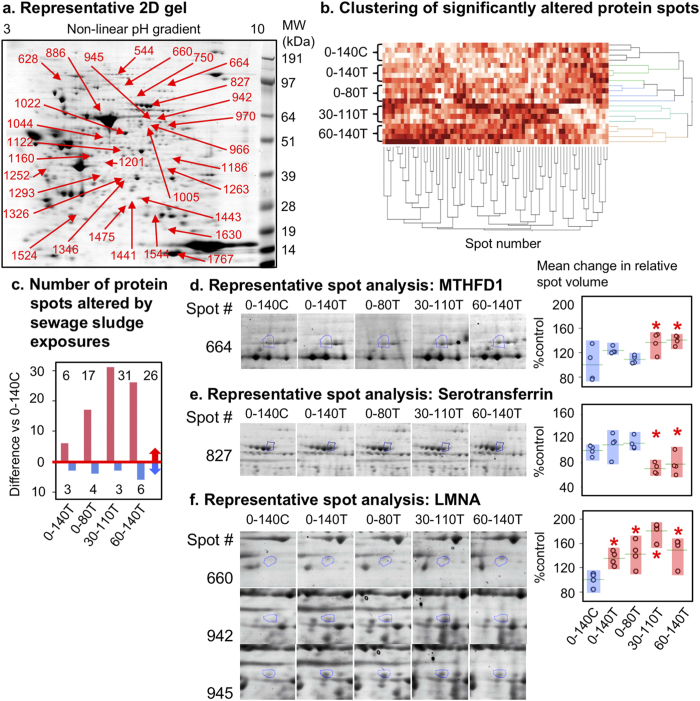
Analysis of differentially expressed fetal ovarian proteins following maternal exposure to sludge during critical developmental stages. (**a**) Representative 2-D gel showing significantly altered proteins excised for identification by LC-MS/MS (Table S5). (**b**) Cluster analysis of altered proteins highlights the separation between the 0–140C, 0–140T and 0–80T groups from the 30–110T and 60–140T groups. Sludge exposure generally increased protein expression across all treatments, particularly in the 30–110T and 60–140T groups (**c**). Representative zoom-boxes and quantification are shown for (**d**) methylenetetrahydrofolate dehydrogenase 1, (**e**) serotransferrin and (**f**) Lamin A/C plus M/C. Each treatment group is shown with the protein circled in blue. Group means indicated by horizontal green lines. The range of control (0–140C) animals is highlighted by a blue bar and those of differing (P < 0.05) treatment groups with an orange bar and a red asterisk.

**Figure 5:**
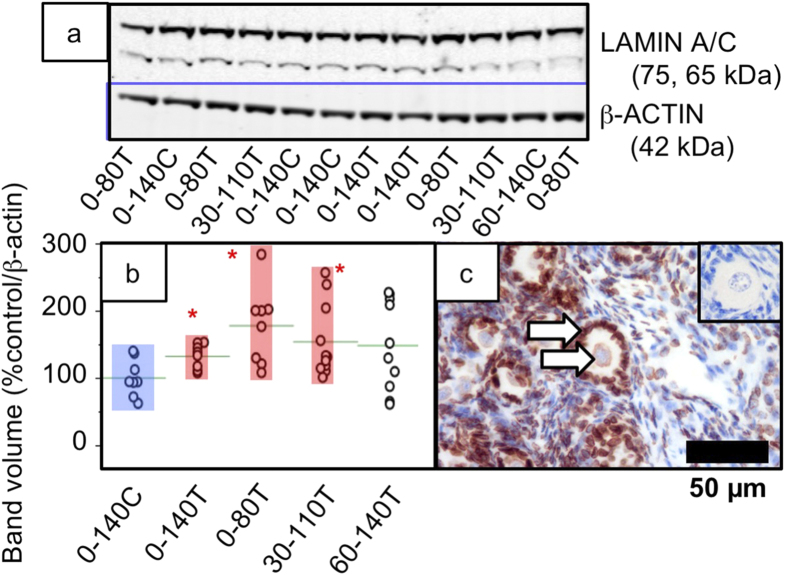
Quantification and localisation of nuclear laminin proteins. (**a**) cropped Western blot for 75, 65 kDa LAMIN A/C and below the blue line, beta-actin load control. The full length images are shown in Supplementary Fig. 3 and gels were run under the same experimental conditions. (**b**) Expression (band volume) of 65 kDa LAMIN C increases in sludge-exposed fetal ovaries. The range of control (0–140C) animals is highlighted by a blue bar and those of significantly differing treatment groups with an orange bar (P < 0.05) and a red asterisk. (**c**) Immunoreactivity for LAMIN A/C is noticeable in nearly every cell but staining is more condensed in pre-granulosa or granulosa cells than in the oocyte or stromal cells.

Previously unused pastures were fertilized with a single treatment of thermally dried sewage sludge in mid-September (2.25 metric tons of dry matter/ha; Treated; T) or with inorganic fertilizer balanced for nitrogen (225 kg/ha/year; Control; C). Experimental animals were maintained on either C or T pastures. Mature Texel ewes were allocated to one of five treatment groups (Fig. 1) randomly within body condition score class, at the beginning of the study. Body condition scoring served as a measure of nutritional state and this was repeated at approximately monthly intervals from the time of first mating through to the day of euthanasia. Each group initially comprised approximately 14 animals but the results reported concern only those carrying at least one female fetus. Ewes of four of the T groups were exposed to sewage sludge-treated pastures from 0–140 days or for an 80 day period during early (0–80 days), mid (30–110 days) or late (60–140 days) gestation (Fig. 1). In order to manage the exposure of ewes in each of the 5 treatment groups as near-contemporaneously as possible, all ewes were mated by Texel rams at the second synchronized estrus following withdrawal of progestagen sponges (Chronolone, 30 mg; Intervet, Cambridge, UK). Pregnant ewes were transferred from the Control pastures to Treated pastures (and back again), as necessary, to achieve exposure to sludge-treated grasslands during the prescribed periods of gestation. The mating times of each group were calculated to ensure that groups of animals at different stages of gestation were exposed to sludge-treated pastures during a similar calendar period (+5 days). When pregnant ewes were removed from the treated pasture, they were maintained for a few days on a separate area of pasture before returning to the control pastures in order to ensure that contamination of the latter, via faeces and urine, was minimized. Animals were condition scored at approximately monthly intervals from the time of first mating to provide an index of nutritional state. However, animals were not fed any supplement during the study so that they were not exposed to any additional EDCs that might be present in such supplements; all ingested nutrients were derived from pasture. Animals of each treatment group were euthanized at Day 140 of gestation, according to Schedule 1 protocols, as defined by the UK Animals (Scientific Procedures) Act (1986). At slaughter, data and tissues were collected and processed as described previously^11^. In brief, fetal anogenital distance (AGD) was measured and fetal ovary weights recorded. Ovaries were either fixed in neutral buffered formalin (NBF) for histological analysis or snap frozen in liquid nitrogen and stored at −80 °C for protein and RNA extraction. Maternal and fetal liver samples were wrapped in aluminum foil and stored at −20 °C until analysis for selected EDCs.

### EDC determinations and hormone assays

Soil samples (approximately 50 cores; 5 cm diameter; 5 cm depth) were collected from the pastures at the beginning of the main period of animal exposure, pooled within plots and analyzed for selected EDCs to provide an index of animal exposure. Soil and liver samples were analysed to determine concentrations of selected polychlorinated biphenyls (PCBs), polybrominated diphenyl ethers (PBDEs), polycyclic aromatic hydrocarbons (PAHs) and diethyl hexyl phthalate (DEHP) as previously described in^9^. Fetal serum levels of progesterone, total testosterone (bound and unbound), free T3 and free T4 were measured as previously described^11^,^13^ with the automated ADVIA Centaur® XP competitive immunoassay system (Siemens Healthcare Diagnostics, Camberley, UK). Progesterone mean intra- and inter-assay CV were 1.9% and 3.2%, respectively, and the assay sensitivity was 0.67 nmol/l. Total serum testosterone mean intra- and inter-assay CV were 4.4% and 6.2%, respectively, and the assay sensitivity was 0.35 nmol/l. Free T3 inter-assay CV was 3.08% and assay sensitivity 0.3 pmol/l. Free T4 mean intra- and inter-assay CV were 4.00% and 2.23% respectively and the assay sensitivity was 1.3 pmol/l.

### Ovarian histomorphology, immunohistochemistry and RNA and protein extraction

After fixing, fetal ovaries were processed as previously described^11^ and analyzed using the Lundy scale^46^ to characterize follicle types. Two tissue blocks were generated per ovary and from each block, two non-consecutive sections (4^th^ and 12^th^) were stained with hematoxylin and eosin. Six fields of view (images) were taken from the periphery of each section at × 200 magnification. Consequently, 24 fields of view were counted for each fetal ovary and results calculated as oocyte follicle density (number/mm^2^). Only those follicles with a visible cross-section of the oocyte nucleus were counted. All other follicle numbers were calculated as percent within each follicle class for each exposure period (Table 2). Within each oocyte/follicle type, health status was recorded using three categories: (i) healthy, (ii) intense nuclear staining (INS) of the oocyte, including only those with nuclei that were clearly condensed and compact in comparison to healthy surrounding follicles and (iii) atretic, including oocytes/follicles that had degenerated. In many cases, the cytoplasm had shrunk and was detached from the granulosa cells; degenerated follicles with INS were also recorded as atretic. Although follicles with very intense dark staining of the oocyte nucleus without cytoplasm shrinkage were considered abnormal, the relationship to atresia is uncertain. Consequently, such follicles were characterized differently.

Immunohistochemistry was performed as described, previously^47^. Two retrieval methods were used to expose antigens that may have been masked during fixation of the tissue: a citrate- based (pH 6.0) solution or an EDTA-based (pH 8.8) solution. The primary antibody incubation period was 30 min. Where applicable, the Bond DAB Enhancer (Leica Microsystems Newcastle Ltd.) was used to maximize clarity by changing the color of the diaminobenzidine (DAB) reaction deposit. Antibodies used were (i) Lamin A/C 1/200, mouse (ab49721, Abcam); (ii) UCHL1, 3 μg/ml, rabbit (LS-B489/9526, Lifespan Biosciences), (iii) PD1A3 (ERP57); 1:1000 rabbit (ab13507, Abcam); (iv) TAGLN, 1 μg/L, goat (AHP2034, ABD Serotec) (v) GSTM3, 1:100 rabbit (15214-1-AP, ProteinTech), (vi) GSN, 1 μg/ml mouse (ab55070, Abcam), (vii) HSPA9 (GRP75), 1:200, rabbit (ab53098, Abcam), (viii) COPS4, 1:25 rabbit (HPA036894, Sigma), (ix) BLVRA, 1:100 rabbit (H00000645-A01, Abnova). Sections were visualized with Bond Refine DAB (Leica Microsystems Newcastle Ltd.), washed in water, and counterstained with hematoxylin. IgG-negative control sections, exposed to non-immune serum of the relevant species in the absence of primary antibody, included in all immunohistochemistry runs showed no positive immunostaining.

### Fetal ovarian RNA and protein extraction

RNA and proteins were extracted from ovaries with a Qiagen AllPrep DNA/RNA/Protein mini kit (Qiagen Ltd., Crawley, UK). Tissues were homogenized following manufacturer’s instructions with two main modifications: i) the addition of protease inhibitors (Protease Inhibitor Cocktail, Sigma-Aldrich Company Ltd, Gillingham, UK) to the lysis buffer (RLT) to maximize protein integrity and yield and ii) the re-suspension of protein pellets in Modified Reswell Solution^11^. RNase-free DNase I digestion of the RNA was performed following manufacturers’ recommendations (Qiagen Ltd).

### Customized ovine microarray and quantitative PCR analysis of gene expression

Transcriptome analysis was conducted on a custom 15K Agilent oligo sheep microarray. The catalogue Sheep Gene Expression Microarray 8 × 15K (G4813A-019921) was modified by adding new target sequences and removing redundancies. Briefly, redundancies were identified according to sheep ESTs assembly and oligo annotations performed by Sigenae (http://www.sigenae.org/). Sheep contigs targeted by several different oligos were identified and the best annotated oligos located on the 3′ end of the contig were conserved (a maximum of 2 per contig); the other oligos targeting the same transcript were removed from the array. A total of 1,500 new targets were then added to the array and oligos were designed according to Agilent “GE Probe Design” eArray workflow and tools (https://earray.chem.agilent.com/earray/) using the default options (Base Composition Methodology, one oligo par target, 60bp length, 3′ Bias Design). The array was enriched with transcripts isolated and identified in the fetal developing sheep ovary by subtractive cDNA library construction (SSH)^48^ and completed with control genes known to be expressed in the female and male developing gonads. New oligos added to the array as well as native Agilent oligos maintained on the array were annotated using the Sigenae SigReannot tool^49^. The final version of annotation showed that the array contained oligos which can be linked to about 7,500 different genes. Labeling and hybridization were performed at the “Plate-forme Biopuces et Sequençage” (http://www-microarrays.u-strasbg.fr/). RNA integrity and quantity was checked, respectively, by Bioanalyzer (Agilent) analysis and Nanodrop ND-1000 UV measurement. Labeling and hybridization were carried out using the Quick Amp Labeling kit for one-color labeling (Agilent, 5190-0442) and the One-Color RNA Spike-in Kit (Agilent, 5188–5282) according to the manufacturer’s instructions with 200 ng of total RNA starting quantities. Arrays were scanned with the Agilent DNA Microarray Scanner Model G2565B and image analysis performed with Agilent Feature Extraction software v9.5.3.1. In order to verify the quality and hybridization reproducibility of the array, hybridization tests with ovary RNAs from control animals were performed using one-color labelling and standard hybridization: 70–80% of spots were significantly hybridized and replicate experiments showed good reproducibility.

Confirmatory transcript expression by real-time PCR was determined as previously described^47^. In brief, RNA was treated using RQ1 RNase-free DNase (Promega, Southampton UK) prior to cDNA synthesis using the Transcriptor First Strand cDNA Synthesis Kit (Roche, Welwyn Garden City, UK) following the manufacturers instructions. Standard curves were generated using 1:5 serial dilutions of pooled cDNA. The cDNA samples were diluted 1:50 in dH_2_O prior to analysis by qPCR. Oligonucelotide primers and hybridisation probes were designed using Primer3 software. The DNA probes were synthesised with a 5′-FAM fluorophore and a 3′-TAMRA quencher (Eurofins, Ebersberg, Germany). Genbank accession numbers and probe and primer sequences are listed in Supplementary Table S8.

All qPCR samples were run in triplicate and included a no temple control. qPCR was conducted using the LightCycler^®^ 480 Probes Master (Roche) according to the manufacturers instructions. The 20 μl reaction volumes contained 1 × Probes Master mix, 0.5 μM of each primer, 0.1 μM probe and 5 μl cDNA sample. The thermal cycling conditions were 10 minutes at 95 °C (initial denaturing), 45 to 55 cycles of 15 seconds at 95 °C, 30 seconds at 60 °C and 1 second at 72 °C (data acquisition). The housekeeping genes GAPDH, HPRT and YWHAZ were tested for stability using geNorm, NormFinder and ANOVA analysis. The qPCR data was analysed using Roche LightCycler480 software and normalised using the geNorm method.

### Validation of transcriptomic data by qPCR

Transcripts differentially expressed by array in the 0–140 day exposure group compared to controls were ordered on the basis of greatest positive and negative fold change. Nine genes, including the two most differentially expressed transcripts, were selected to validate the changes observed using the microarray. Only transcripts coded by well-annotated genes were selected for this purpose.

### Proteomics and Western blotting

Within each treatment group, equal amounts of protein from each ovary extract were combined to construct a single protein “pool” for each treatment group. The four protein pools were treated with ReadyPrep 2-D Cleanup Kit (Bio-Rad Laboratories Ltd, Hemel Hempstead, UK) to remove salt and other contaminants according to the manufacturer’s instructions. Equal amounts (between gels and groups) of the soluble protein fractions were separated using 2D gel electrophoresis in quadruplicate (n = 4) as described^10^. The pools represent the biological variability between individuals while the quadruplicate gels represent the technical variability^10^,^11^,^34^. The gels were analysed using Progenesis SameSpots software, V 6.01 (Nonlinear Dynamics, Newcastle, UK) which combine the gel quadruplicates and calculated fold-changes and significance (by ANOVA of log-normalized values). Differentially expressed protein spots were selected for spot cutting once these ANOVA values had been verified (see Statistical Analysis below). Molecular masses and pIs of spots of interest were estimated from separate gels electrophoresed with pH and MW markers. Proteins in the gel pieces were digested with trypsin and analyzed (see^11^ for details). Mass lists in the form of Mascot Generic Files were created automatically and used as the input for Mascot MS/MS Ions searches of the NCBInr database using the Matrix Science web server (www.matrixscience.com). The default search parameters used were: Enzyme = Trypsin, Max. Missed cleavages = 1; Fixed modifications = Carbamidomethyl (C); Variable modifications = Oxidation (M); Peptide tolerance ± 1.5 Da; MS/MS tolerance ± 0.5 Da; Peptide charge = 2+ and 3+; Instrument = ESI-TRAP. Statistically significant MOWSE scores and good sequence coverage were considered to be positive identifications.

Individual ovary protein extracts were electrophoresed (30 μg protein/lane) on 26-lane 1-DE 4–12% Bis-Tris gels (Invitrogen Ltd, Paisley, UK) under reducing conditions (MOPS buffer, Invitrogen) and transferred to Immobilon™-FL membranes (Millipore (UK) Ltd, Watford, UK) as described previously^10^,^11^. Odyssey Two-Colour Protein Molecular Weight Markers (LI-COR Biosciences UK Ltd, Cambridge, UK) were electrophoresed in 3 lanes of every gel. The membranes were blocked (1 hr, room temperature) with Odyssey Blocking Buffer (927–4000: LICOR + PBS) and were incubated with primary anti-lamin A/C (mouse 1:5,000, AbCam AB49721) (diluted in blocking buffer + PBST) at 4 °C overnight. An anti-ACTB (β-Actin) load control of differing species was used (mouse 1:5,000, AbCam AB8226 or rabbit 1:10,000, Sigma-Aldrich A5060). IRDye^®^ infrared secondary antibodies were obtained from LI-COR. Protein bands were visualized using the LI-COR Odyssey® Infrared Imaging System and the resulting electronic images were analyzed using TotalLab TL120 software (v2008.1; Nonlinear Dynamics Ltd, Newcastle-upon-Tyne, UK) to determine the molecular weights and band volumes. The band volumes of ACTB (β-Actin) were compared between treatment groups to check the validity of this load control for fetal ovaries from the 4 treatment groups and the controls.

### Statistical and array analyses

Unless otherwise stated, data are presented as mean ± s.e.m. Fold-changes presented as positive or negative values are indicative of an increase or decrease, respectively, relative to controls. Data analyses were performed using JMP 7 (Thomson Learning, London, UK). Normality of data distribution was tested with the Shapiro-Wilk test and non-normally distributed data were log or arcsine transformed, the latter approach relating to proportional data. Following transformation, the assumptions of the statistical tests were met (normality and homogeneity of variance of residuals). Values for control and sewage sludge-exposed groups were compared using one-way ANOVA for morphological and endocrine data, 1-DE Western Blot band volumes (normalized relative to ACTB expression separate for each lane) and normalized spot volumes (% of total spot volume for each gel separately). A linear mixed model was used to account for litter size effects [fixed effects: number of fetuses and treatment, random effect: ewe]. Tissue EDC concentration data were log_10_ transformed prior to analysis. Mean concentrations were compared using ANOVA, with the main effects being treatment and age (maternal vs fetal). For gene array, data processing and analysis were conducted using Biocondutor packages suite (http://www.bioconductor.org/index.html) and LIMMA package^50^ with the R statistical program. Raw median signal from Feature Extraction array files was used as non-processed signal and log2 transformed. Background was then subtracted locally and intra-array normalization performed by subtracting the array median signal from each spot signal on the same array. Multiple testing corrections were applied [Benjamini-Hochberg method^51^] and differentially expressed transcripts were considered under a False Discovery Rate (FDR) of 5%. Functional analysis was conducted on transcripts and proteins that exhibited statistically significant, differential expression, using Ingenuity Pathway Analysis (IPA, http://www.ingenuity.com/).

## Additional Information

**How to cite this article**: Lea, R. G. *et al.* The fetal ovary exhibits temporal sensitivity to a ‘real-life’ mixture of environmental chemicals. *Sci. Rep.* **6**, 22279; doi: 10.1038/srep22279 (2016).

## Supplementary Material

Supplementary Information

## Figures and Tables

**Table 1:** Morphology and endocrinology of Day 140 female fetuses following exposure to sludge treated pastures.

	Exposure Groups				
	0–140C	0–140T	0–80T	30–110T	60–140T
Morphology					
No. Ewes carrying ≥ 1 female fetus^A^	8	5	6	7	7
No of female fetuses^B^	9	8	9	11	10
Fetal weight (kg)	**5.01 ± 0.20**^**a**^	4.59 ± 0.32	**4.29 ± 0.25**^**b**^	**4.29 ± 0.27**^**b**^	**3.59 ± 0.33**^**b**^
*Body weight-normalized parameters (/kg)*					
AGD^C^ (mm/kg)	**2.0 ± 0.2**^**a**^	1.7 ± 0.1	1.9 ± 0.2	**3.3 ± 0.2**^**b**^	**3.1 ± 0.3**^**b**^
Ovary (mg/kg)	16.6 ± 2.0	20.1 ± 3.2	19.4 ± 2.6	14.3 ± 0.9	17.5 ± 1.2
Thyroid (mg/kg)	**221 ± 18**^**a**^	223 ± 20	227 ± 17	265 ± 17	**308 ± 26**^**b**^
Adrenals (mg/kg)	125 ± 12	115 ± 3	110 ± 8	128 ± 9	156 ± 14
Uterus (mg/kg)	**212 ± 10**^**a**^	233 ± 16	244 ± 19	217 ± 15	**162 ± 11**^**b**^
Liver (g/kg)	**24.3 ± 0.4**^**a**^	**20.5 ± 1.1**^**b**^	24.2 ± 1.2	24.9 ± 1.1	**28.4 ± 1.7**^**b**^
Endocrinology					
Testosterone (nmol/l)	**2.7 ± 0.1**^**a**^	3.2 ± 0.3	**3.5 ± 0.3**^**b**^	2.7 ± 0.3	2.3 ± 0.1
Progesterone (nmol/l)	119 ± 11	102 ± 20	136 ± 18	90 ± 11	132 ± 13
Free T3 (pmol/l)	**2.7 ± 0.2**^**a**^	3.4 ± 0.4	3.0 ± 0.3	3.2 ± 0.2	**1.7 ± 0.2**^**b**^
Free T4 (pmol/l)	**25.3 ± 1.3**^**a**^	26.3 ± 2.2	26.7 ± 1.4	27.1 ± 1.4	**29.5 ± 1.0**^**b**^

^A^75% of ewes (25/33) carried twin pregnancies. The remaining pregnancies were singleton (n = 3) and triplet (n = 5).

^B^Fetuses were used from all pregnant ewes (≥1 per ewe).

^C^AGD = anogenital distance. Values represent mean ± SEM. Experimental groups: C = Control, T = Exposed. Differing superscripts indicate differences relative to 0–140C at P < 0.05. Paired organs were combined into a single weight prior to normalization against fetal weight.

**Table 2:** Effects of sewage sludge chemicals on relative proportions of ovarian oocyte/follicle types in day 140 fetal ovaries.

Exposure Description	Oocyte/follicle proportions (%total oocytes/follicles/follicle class)				
	0–140C None	0–140T Continuous	0–80T Early	30–110T Mid	60–140T Late
No. ovaries assessed	5	8	9	10	9
Type* 0 (n/mm^2^)	0.35 ± 0.20	1.02 ± 0.42	1.05 ± 0.36	1.57 ± 0.51	0.68 ± 0.27
	0.35 ± 0.20	1.02 ± 0.42	1.05 ± 0.36	1.57 ± 0.51	0.68 ± 0.27
Type 0 (%)					
Healthy**	**60.7 ± 21.5**^**a**^	**89.8 ± 5.9**^**b**^	71.0 ± 10.8	82.7 ± 9.7	63.3 ± 10.1
INS	5.3 ± 3.3	7.1 ± 4.6	10.0 ± 4.3	8.0 ± 5.0	9.9 ± 3.4
Atretic	34.0 ± 22.7	3.2 ± 2.1	10.0 ± 10.4	9.3 ± 4.9	26.9 ± 8.8
Type 1 (n/mm^2^)	3.87 ± 2.61	4.73 ± 1.76	5.81 ± 1.78	7.29 ± 1.83	9.85 ± 4.14
Type 1 (%)					
Healthy	**70.1 ± 23.4**^**a**^	87.2 ± 2.4	92.2 ± 1.5	88.4 ± 2.2	**92.4 ± 1.8**^**b**^
INS	0.6 ± 0.4	6.6 ± 1.7	6.0 ± 1.5	5.9 ± 2.5	2.9 ± 0.9
Atretic	**29.3 ± 23.6**^**a**^	**6.2 ± 1.8**^**b**^	**1.9 ± 0.5**^**b**^	**5.7 ± 1.3**^**b**^	**4.7 ± 1.5**^**b**^
Type 1a (n/mm^2^)	11.86 ± 4.57	11.67 ± 1.98	11.53 ± 2.79	18.24 ± 2.77	19.56 ± 3.42
Type 1a (%)					
Healthy	**98.0 ± 1.0**^**a**^	**91.6 ± 2.2**^**b**^	**89.3 ± 1.5**^**b**^	**86.7 ± 2.5**^**b**^	**91.1 ± 1.2**^**b**^
INS	**0.7 ± 0.3**^**a**^	3.0 ± 1.2	**3.8 ± 0.8**^**b**^	**3.7 ± 1.1**^**b**^	2.9 ± 0.4
Atretic	**1.3 ± 0.8**^**a**^	5.5 ± 1.5	**6.9 ± 1.2**^**b**^	**9.6 ± 1.8**^**b**^	**6.0 ± 0.9**^**b**^
Type 2 (n/mm^2^)	**1.66 ± 0.29**^**a**^	1.85 ± 0.54	1.11 ± 0.21	2.29 ± 0.37	**4.67 ± 0.83**^**b**^
Type 2 (%)					
Healthy	**96.0 ± 1.1**^**a**^	85.5 ± 4.8	93.5 ± 2.1	**80.4 ± 5.0**^**b**^	88.4 ± 3.8
INS	**1.0 ± 0.7**^**a**^	1.2 ± 0.8	1.5 ± 0.8	**5.3 ± 0.8**^**b**^	1.2 ± 0.4
Atretic	3.0 ± 1.0	13.3 ± 4.5	5.1 ± 2.2	14.4 ± 4.2	10.4 ± 3.9
Types 3–5 (n/mm^2^)	0.17 ± 0.06	0.17 ± 0.05	0.22 ± 0.06	0.20 ± 0.06	0.17 ± 0.06
Types 3–5 (%)***					
Healthy	100	100	95.7 ± 4.3	90.6 ± 3.9	96.2 ± 2.7
INS	0	0	0	2.4 ± 1.6	0
Atretic	0	0	4.3 ± 4.3	7.0 ± 3.9	3.8 ± 2.7

Oocyte/follicle proportions are expressed as a % relative to oocyte/follicle density within each follicle class, at different times of gestation. Proportions are shown according to developmental type* and health category**; values represent mean ± SEM, differing superscripts indicate statistically significant differences vs Control (0–140 C) at p values < 0.05. *Developmental types: type 0 = isolated or naked oocyte (without associated granulosa cells (GC)). Follicle types (Lundy *et al.*^46^): type 1 = primordial follicle (oocyte with flattened GC; type 1a =Transitory/activated follicle (a mixture of flattened and ≥1 cuboidal GC; type 2 = primary follicle (1 to <2 complete layers of cuboidal GC; type 3 = small pre-antral follicle (2 to <4 complete layers of cuboidal GC); type 4 = large pre-antral follicle (4 to <6 layers of cuboidal GC); type 5 = small antral follicle (>5 layers cuboidal GC).**Health categories: Healthy, INS = intense nuclear staining and compact oocyte nucleus, Atretic = degenerated. ***Types 3–5 combined because of low numbers. P = 0.055 vs control.
