# Supplementary material for: The fetal ovary exhibits temporal sensitivity to a ‘real-life’ mixture of environmental chemicals
Source: Sci Rep. 2016 Mar 2;6:22279. doi: 10.1038/srep22279 (PMC4773987; doi:10.1038/srep22279)
Supplement: Supplementary Information [file srep22279-s1.pdf]

Supplementary Information

The fetal ovary exhibits temporal sensitivity to a 'real-life' mixture of environmental chemicals

Richard G. Lea[1↑*], Maria R. Amezaga[2,5↑], Benoit Loup[3↑], Beatrice Mandon-Pépin[3], Agnes Stefansdottir[2,6,↑], Panagiotis Filis[2], Carol Kyle[4], Zulin Zhang[4], Ceri Allen[1], Laura Purdie[1], Luc Jouneau[3], Corinne Cotinot[3,↑], Stewart M. Rhind[4,↑↑], Kevin D. Sinclair[1↑]and Paul A. Fowler[2↑].

1.Schools of Veterinary Medicine and Biosciences, University of Nottingham, Leicestershire, LE12 5RD, UK.

2. Institute of Medical Sciences, School of Medicine, Medical Sciences & Nutrition, University of Aberdeen, Foresterhill, Aberdeen, AB25 2ZD, UK;

3.UMR,, Biologie du Développement et Reproduction, INRA, Universite Paris Saclay, 78350, Jouy-en-Josas, France;

4.The James Hutton Institute, Craigiebuckler, Aberdeen, AB15 8QH, UK;

5. Present address: Research and Development Office, NHS Grampian, Foresterhill, Aberdeen, AB25 2ZB

6. Present address: Centre for Integrative Physiology, University of Edinburgh, EH8 9XD, UK

[↑] These authors contributed equally to the manuscript

[↑↑] Stewart Rhind deceased March 2013

[*]Corresponding author: richard.lea@nottingham.ac.uk

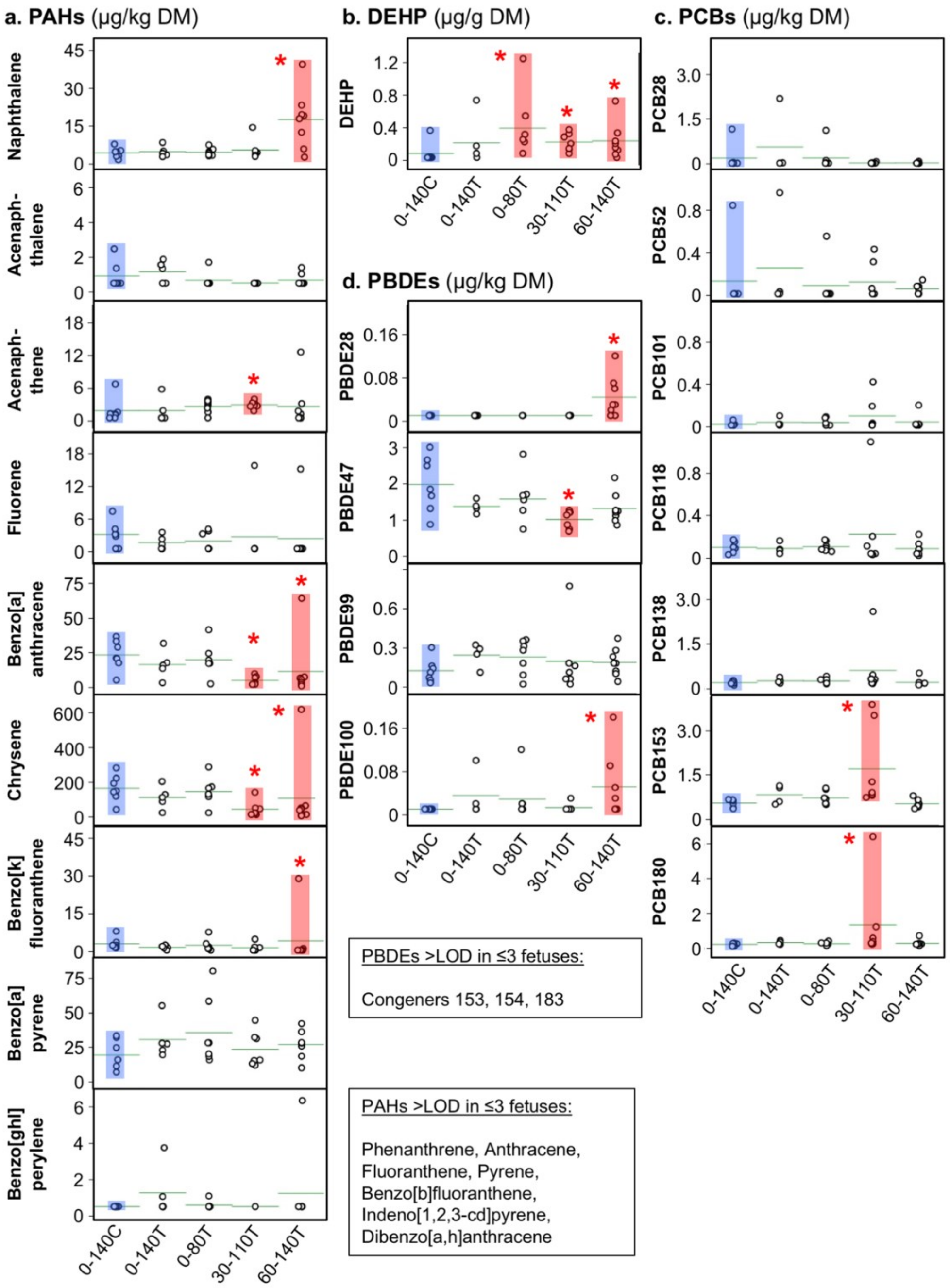

Supplementary figure S1. Maternal hepatic concentrations of 21 environmentally-relevant ECs after sewage sludge exposure. (A) polycyclic aromatic hydrocarbons (PAHs), (B) Di(2-ethylhexyl)phthalate (DEHP), (C) Polychlorinated biphenyls (PCB), (D) Polybrominated Diphenyl Ethers (PBDE). Values for individual livers are shown by circles and the group means by horizontal green lines. The range of control (0-140C) animals is highlighted by a blue bar and those of significantly differing treatment groups with an orange bar ($P<0.05$) and a red asterisk. Where EDC levels were only above the limit of detection (LOD) in 3 or fewer fetuses, the data are not shown (text box).

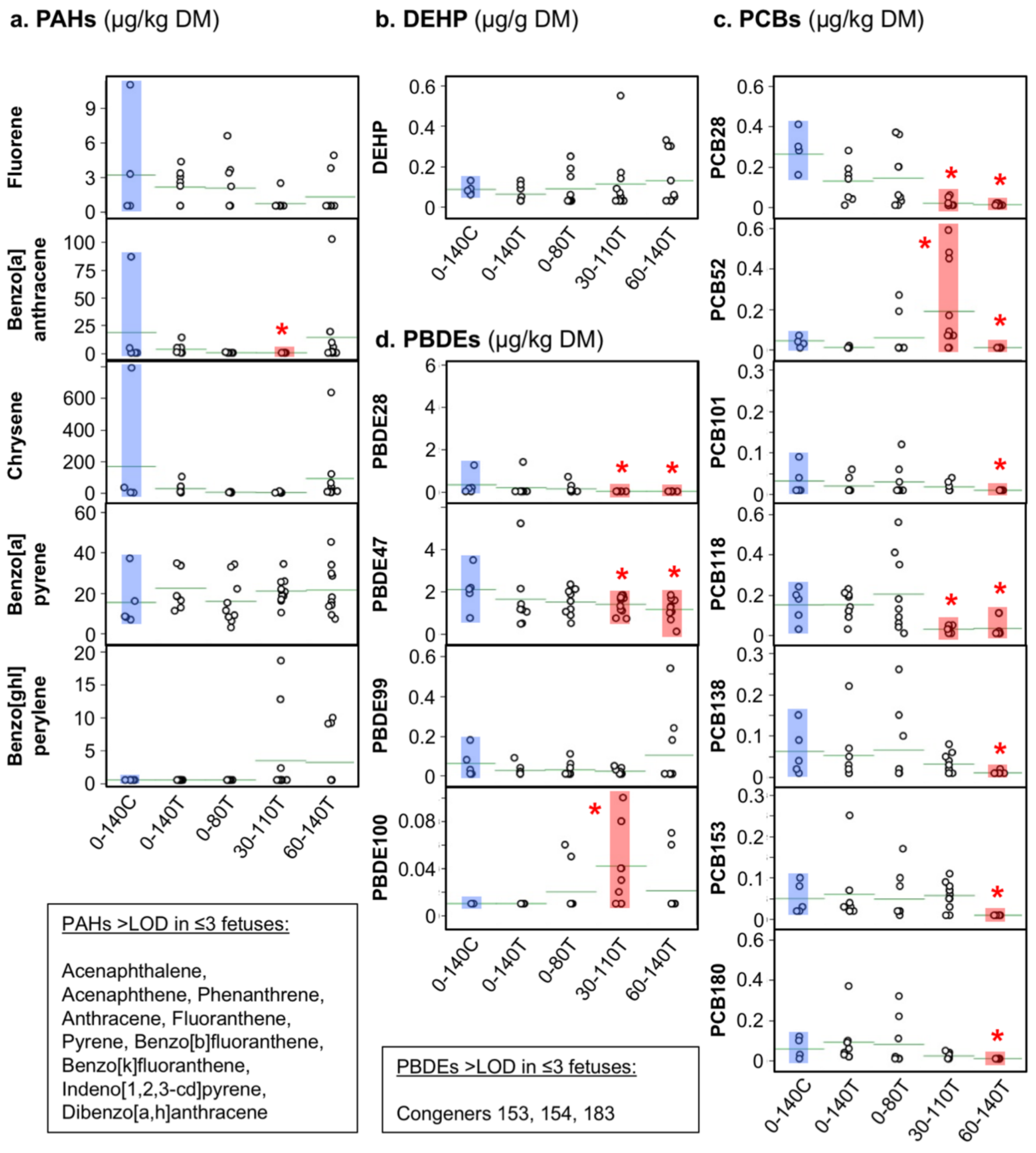

Supplementary figure S2. Fetal hepatic concentrations of 24 environmentally-relevant ECs after sewage sludge exposure. (A) polycyclic aromatic hydrocarbons (PAHs), (B) Di(2-ethylhexyl)phthalate (DEHP), (C) Polychlorinated biphenyls (PCB), (D) Polybrominated Diphenyl Ethers (PBDE). Values for individual fetuses are shown by circles and the group means by horizontal green lines. The range of control (0-140C) animals is highlighted by a blue bar and those of significantly differing treatment groups with an orange bar (P<0.05) and a red asterisk. Where EDC levels were only above the limit of detection (LOD) in 3 or fewer fetuses, the data are not shown (text box).

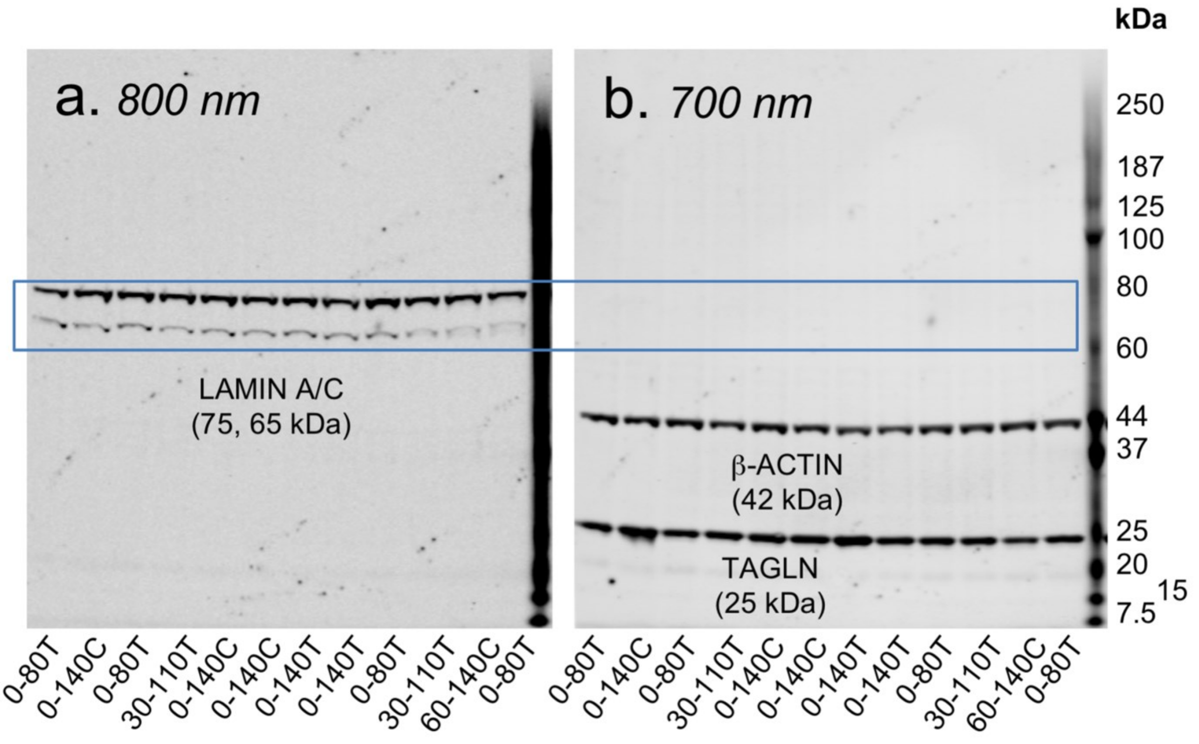

Supplementary Figure S3. Full length images (reported in Fig. 5) from a single Western blot membrane probed for (a) LAMIN A/C and (b) β-ACTIN as a load control. The two frequencies used by the Odyssey imaging system are shown separately rather than merged for clarity. The protein TAGLN was also probed prior to the addition of anti-β-ACTIN but showed no statistically significant difference between treatment groups and was not reported in the main results.

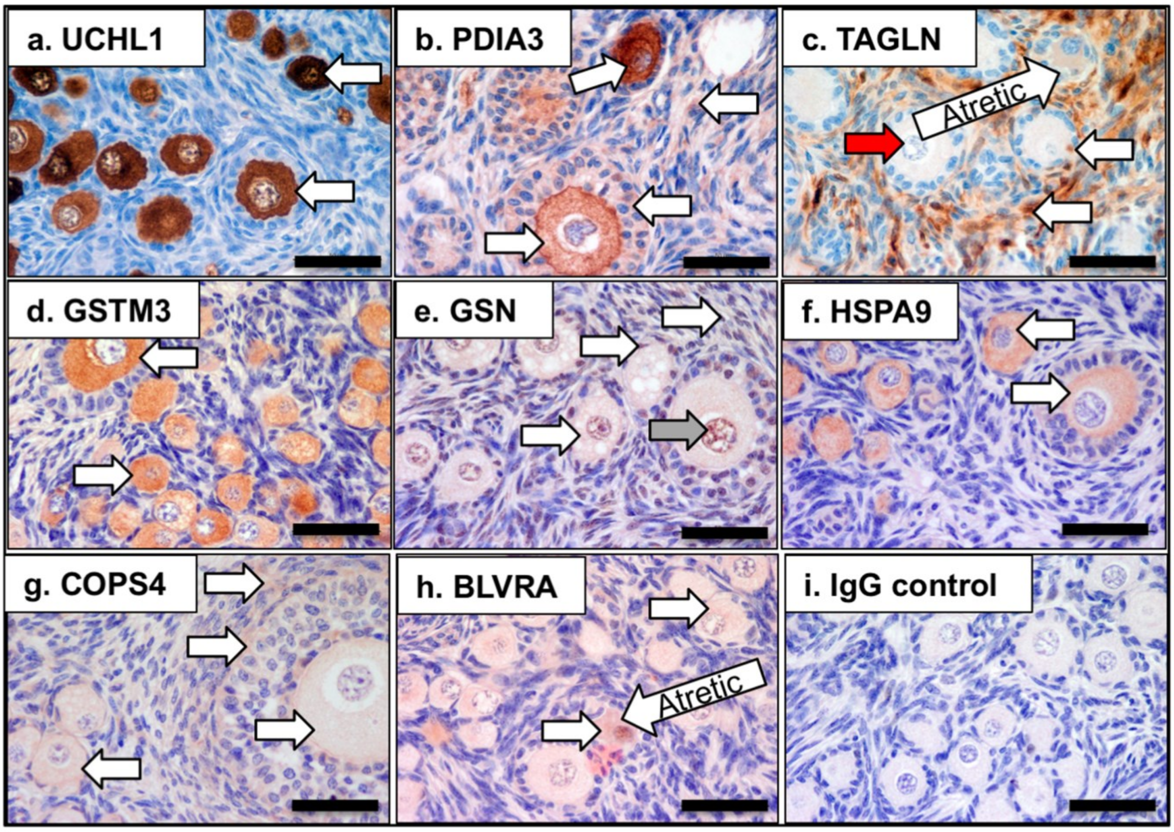

Supplementary figure S4. Immunolocalisation of proteins with altered expression in Day 140 fetal ovaries exposed to sewage sludge at different stages of gestation. Positive staining is brown (DAB), counterstained by hematoxylin (blue). The bars denote scale for each image separately. Plain white arrows highlight immuno-positive cells. Colored or labeled arrows denote features of interest. UCHL1 (A), GSTM3 (D) and HSPA9 (F) were localized to the cytoplasm of the oocyte. HSPA9 (F) was also visible in the granulosa cells. PDIA3 (B) was principally localized to the oocyte cytoplasm, but also showed some expression in granulosa cells. TAGLN (C) was localized to somatic cells, especially mesenchymal cell streams although a few probable granulosa cells were also immune-positive. GSN (E) was weakly localized to oocyte cytoplasm and to some granulosa and somatic cells. Some staining of nuclei was apparent. COPS4 (G) was widely but weakly localized to the cytoplasm of oocytes, granulosa cells and some somatic cells around the follicles. In contrast, BLVRA (H) was weakly localized principally to oocyte cytoplasm although heavier staining of some atretic follicles was observed. In all cases IgG-negative slides incubated with non-immune serum of the appropriate species were characterized by an absence of brown stain (I). Scale bar = 50μM.

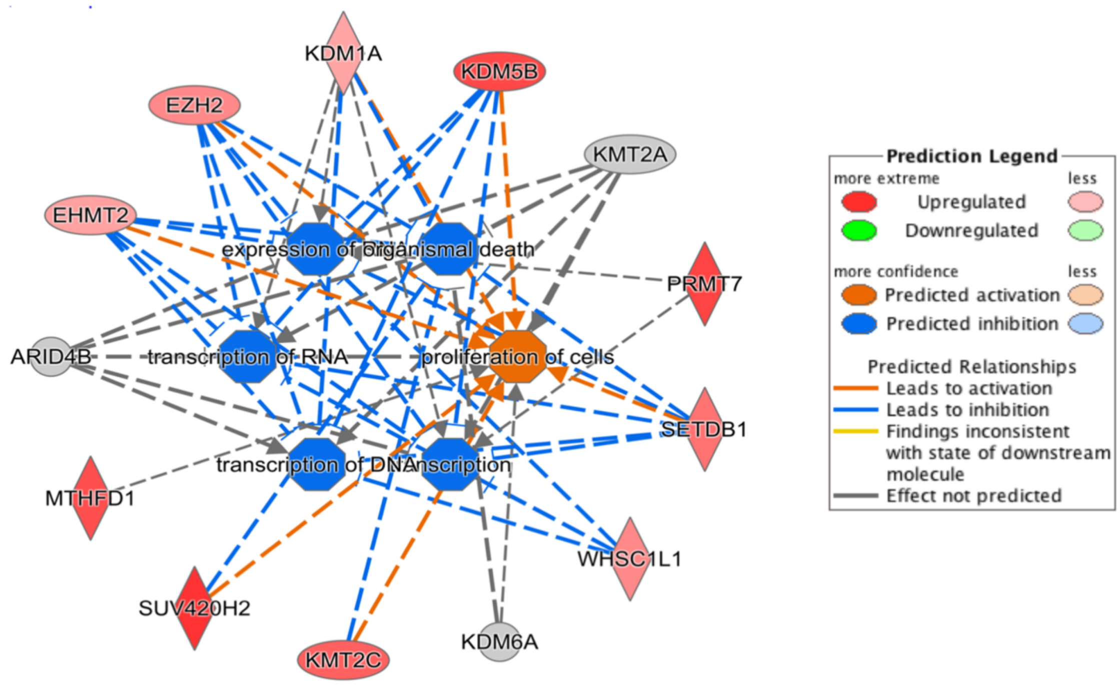

Predicted downstream effects of the methylation-associated protein and genes affected in the day 30-110 and 60-140 exposures groups (Supplementary Tables 2 & 5) analysed together using IPA. The Activation z-score predicts the activation state of the upstream regulator, using the gene expression patterns of the genes downstream of an upstream regulator. An absolute z-score of ≥ 2 is considered significant.

Diseases or Functions	Predicted Activation State	Activation z-score	Molecules	# Molecules
Organismal death	Decreased	-2.401	ARID4B,EHMT2,EZH2,KDM1A,KDM6A,KMT2A, KMT2C,SETDB1,SUV420H2	9
Transcription of DNA	Decreased	-2.263	ARID4B,EHMT2,EZH2,KDM1A,KDM5B,KMT2A,SETDB1,WHSC1L1	8
Transcription	Decreased	-2.193	ARID4B,EHMT2,EZH2,KDM1A,KDM5B,KMT2A,PRMT7,SETDB1,WHSC1L1	9
Expression of RNA	Decreased	-2.193	ARID4B,EHMT2,EZH2,KDM1A,KDM5B,KMT2A,PRMT7,SETDB1,WHSC1L1	9
Transcription of RNA	Decreased	-2.193	ARID4B,EHMT2,EZH2,KDM1A,KDM5B,KMT2A,SETDB1,WHSC1L1	8
Proliferation of cells	Increased	2.530	ARID4B,EHMT2,EZH2,KDM1A,KDM5B,KDM6A, KMT2A,KMT2C,MTHFD1,SETDB1,SUV420H2	11

Supplementary figure S5. Networks affected by sewage sludge exposure based on epigenetic effects on the proteome (Supplementary table 5: MTHFD1) and transcriptome (Supplementary table 3: EZH2, AOF2, ARID4B, EHMT2, KDM5B, MLL, MLL3, PRMT7, SETDB1, SETDB2, SUV420H2, UTX, WHSC1L1) of the 30-110 and 60-140 groups combined compared with control (0-140C). Analysis was performed using IPA.

Supplementary Table S1: Effects of sludge exposure on soil concentrations of individual polycyclic aromatic hydrocarbons (PAH).

**PAH in Soil (µg/kg of dry matter)**			**Changes in PAH relative to Controls**	
	Control[A] n=1	Treated[A] n=3[C]	Maternal liver	Fetal Liver
Naphthalene	23.1	80.6 ± 22.3	↑60-140	<LOD[B]
Acenaphthalene	5.1	8.6 ± 4.2	No Change	<LOD
Acenaphthene	11.9	27.4 ± 12.0	↑30-110	<LOD
Fluorene	10.8	36.9 ± 15.3	No Change	No Change
Benzo[a]anthracene	121.7	235.6 ± 84.1	↓30-110, ↓60-140	↓30-110
Chrysene	192.1	310.3 ± 112.3	↓30-110, ↓60-140	No Change
Benzo[k]fluoranthene	126.3	170.1 ± 63.6	↑60-140	<LOD
Benzo[a]pyrene	130.6	163.4 ± 57.7	No Change	No Change
Benzo[ghi]perylene	54.5	135.6 ± 61.1	No Change	No Change

[A]Soil samples were taken in December from 1 control and 3 sewage sludge treated pastures.
[B]Below limit of detection, [C]Mean ± SEM

Supplementary table S2: Fetal ovarian genes altered by sewage sludge exposure of ewes:

(A) Ovarian genes altered following exposure throughout gestation (0-140 days) or during different periods of gestation: early (0-80 days), mid (30-110 days) and late (60-140 days) gestation.

0-140C vs 0-140T (33 differential probes):

	Agilent Probe Name	Target EST Accession	Ensembl Gene ID	Gene Symbol	Log Fold Change
Up-regulated genes	A_70_P001791	EE768843, NR_036644.1	-	RN28S1	1.84
	BL_UN4_F000144	CD288357, NR_036644.1	-	RN28S1	1.83
	BL_OCA_X000661	EE800761, NR_036644.1	-	RN28S1	1.24
	BL_OTH_F000135	AJ874675	MHC class I antigen, Multiple targets		1.22
	A_70_P068241	AJ874684, EF489531	ENSBTAG00000018701	LOC616942	1.01
	A_70_P070391	EE818692	ENSBTAG00000017021	MCM4	0.96
	A_70_P057546	EE830287	ENSBTAG00000011595	IHPK1	0.94
	BL_OCA_X000220	EE747067	ENSBTAG00000000836	P05786	0.87
	A_70_P037711	EE773783	ENSBTAG00000002728	ARID1B	0.85
	BL_UN4_R000141	CD286372	ENSBTAG00000014490	BAT1	0.82
	A_70_P040451	EE828873	ENSBTAG00000004899	ABLIM	0.82
	A_70_P021386	EE829899	ENSBTAG00000000599	CCNI	0.79
	A_70_P021286	EE751423	ENSBTAG00000021880	CABC1	0.76
	A_70_P007651	EE820840	ENSBTAG00000005064	WARS2	0.75
	A_70_P071491	EE821931	ENSBTAG00000002389	MED19	0.73
	A_70_P007676	EE822384	ENSBTAG00000016448	ZBTB40	0.72
	BL_OCA_R000130	CU653858	ENSBTAG00000008022	PTGFRN	0.72
	BL_OCA_X000861	EE750663	ENSBTAG00000015114	CALR	0.72
Down-regulated genes	BL_OCA_X000085	EE863690	ENSBTAG00000001403	PRKCI	-0.71
	A_70_P025386	FE022182	ENSBTAG00000026307	ZNF629	-0.72

	BL_OCA_F000886	S80867	ENSBTAG00000014567	MYLK	-0.72
	A_70_P030096	NM_001009744, AF254119	ENSBTAG00000016947	AGTR1	-0.73
	A_70_P007591	FE021553	ENSBTAG00000017085	AGA	-0.78
	A_70_P010686	EE755709	ENSBTAG00000010529	FZD6	-0.78
	A_70_P014071	FE025905	-	-	-0.90
	A_70_P033336	DY486945	ENSBTAG00000012638	S100A12	-1.03
	A_70_P066546	FE035791	ENSBTAG00000003747	PTGR2	-1.03
	A_70_P011801	FE030747	-	-	-1.16
	BL_OTH_R000040	CF116541	ENSBTAG00000007900	FIS1	-1.16
	A_70_P008296	CN821983	ENSG00000185222	WBP5	-1.19
	A_70_P026671	EE748059, EE746827, EE750802	ENSG00000211895	IGHA1	-1.46
	BL_OTH_R000484	FE028592	ENSBTAG00000001308	LOC522763	-2.02
	A_70_P041321	AF024645	ENSG00000211890	IGHA2	-2.13

0-140C vs 0-80T (4 differential probes):

	Agilent Probe Name	Target EST Accession	Ensembl Gene ID	Gene Symbol	Log Fold Change
**Up-regulated gene**	BL_OTH_F000135	AJ874675	Multiple Targets		1.17
**Down-regulated genes**	A_70_P026671	EE748059, EE746827, EE750802	ENSG00000211895	IGHA1	-1.48
	A_70_P056141	EE835840	-	-	-1.64
	A_70_P041321	AF024645	ENSG00000211890	IGHA2	-2.03

0-140C vs 30-110T (99 differential probes):

	Agilent Probe Name	Target EST Accession	Ensembl Gene ID	Gene Symbol	Log Fold Change
**Up-regulated genes**	A_70_P031731	FE021050	-	-	1.68
	A_70_P069011	EE754635	-	-	1.27
	A_70_P019481	EE828813	ENSBTAG00000007113	TRRAP	1.26

	A_70_P043666	EE794724	ENSBTAG00000009374	PARP2	1.02
	A_70_P063496	EE777917	ENSBTAG00000002609	TMIGD1	0.98
	BL_CTL_F000095	NM_001110098	ENSBTAG00000040202	JY-1	0.96
	BL_OTH_F000135	AJ874675	Multiple Targets		0.95
	BL_UN4_R000112	CU655439	-	-	0.93
	A_70_P061621	EE749065	ENSBTAG00000005810	PDZK1	0.85
	A_70_P061896	EE805549	ENSBTAG00000014595	LIG1	0.84
	A_70_P056847	M34674	ENSBTAG00000002069	P13752	0.82
	BL_OTH_R000570	CU653314	ENSBTAG00000016548	TEX12	0.81
	A_70_P011466	EE783134	-	-	0.81
	BL_OCA_R000236	CU653314	ENSBTAG00000016548	TEX12	0.81
	A_70_P060631	EE853499	ENSBTAG00000030920	A8MWK0	0.80
	A_70_P006731	FE030420	-	-	0.80
	BL_OCA_R000096	CU653146	ENSG00000167634	NLRP7	0.80
	BL_UN4_R000134	EE834952	-	-	0.79
	A_70_P019141	EE812382	ENSBTAG00000010922	TRAPPC2	0.78
	A_70_P017891	EE748738	ENSBTAG00000007079	LCP1	0.76
	A_70_P041246	AF065146	ENSBTAG00000012290	SCNN1B	0.75
	A_70_P038456	CN824507	ENSBTAG00000012393	AGT	0.75
	A_70_P053296	EE829589	ENSBTAG00000023963	RHBDD1	0.74
	BL_OCA_R000007	CU637765	ENSG00000167634	NLRP7	0.74
	A_70_P001091	FE027551	-	-	0.73
	BL_OTH_F001109	EE855194	ENSBTAG00000024219	TRPV6	0.72
	A_70_P012986	EE848845	ENSBTAG00000019460	MOXD1	0.71
	BL_CTL_F000043	CU652732	ENSBTAG00000016272	TEX11	0.71
**Down-regulated genes**	A_70_P018781	EE783518	ENSBTAG00000009584	SAPS3	-0.70
	A_70_P068856	EE792088	ENSBTAG00000010123	APOE	-0.71
	BL_UN4_R000010	CU638079	-	-	-0.71
	A_70_P037101	EE773698	ENSBTAG00000013801	PBX1	-0.71

A_70_P069291	EE823361	ENSBTAG00000026111	LBH	-0.71
A_70_P011496	DY501713	ENSBTAG00000004261	SPON2	-0.71
A_70_P064761	EE794068	-	-	-0.71
BL_OCA_X000467	EE748868	ENSBTAG00000020309	G3BP1	-0.71
BL_OTH_R000161	DY498768	ENSBTAG00000009491*	WDR74	-0.71
A_70_P072016	FE036025	ENSBTAG00000000146	FARP1	-0.72
BL_OTH_F000355	CF116331	ENSBTAG00000016649	CCND2	-0.72
A_70_P008296	CN821983	ENSG00000185222	WBP5	-0.72
A_70_P008616	EE826054	ENSBTAG00000005586	GATM	-0.72
A_70_P042816	EE756509	ENSBTAG00000003825	PTPN12	-0.72
BL_OCA_R000262	EE789197	-	-	-0.72
A_70_P000006	EE752115	ENSBTAG00000000088	ALCAM	-0.73
A_70_P068551	DY507846	ENSBTAG00000019517	ELN	-0.73
A_70_P027011	CN824409	-	-	-0.74
A_70_P017371	EE756026	ENSBTAG00000033487	C4orf46	-0.74
BL_OCA_X000834	EE754453	ENSBTAG00000026836	ABI3BP	-0.74
A_70_P015531	FE024895	ENSBTAG00000003851	CCNL1	-0.74
BL_OCA_X000741	EE833401	ENSBTAG00000019839	LTBP1	-0.74
A_70_P065771	EE758227	ENSBTAG00000010531	CYP1B1	-0.75
BL_OCA_F000079	CU637962	ENSBTAG00000004115	MYLIP	-0.76
A_70_P040696	EE797585	-	-	-0.76
BL_OTH_F000631	DY481166	ENSBTAG00000005077	CXCL12	-0.76
A_70_P005636	EE784504	ENSBTAG00000015844	TFPI2	-0.77
BL_OCA_F000053	CF118379	ENSBTAG00000016420	CTNNB1	-0.77
A_70_P057331	EE827130	ENSBTAG00000003708	SEC23A	-0.77
BL_OCA_X000507	CU652926	ENSBTAG00000027625	SFRP1	-0.78
A_70_P059406	EE793510	ENSBTAG00000030435	PNRC2	-0.78
A_70_P013441	DY492813	ENSBTAG00000014874	FYTTD1	-0.79
A_70_P007006	EE778135	ENSBTAG00000012615	ZEB2	-0.79

A_70_P046971	EE874463	ENSBTAG00000019472	NR3C1	-0.79
A_70_P048981	EE772304	-	-	-0.79
BL_UN1_R000020	CU653226	ENSBTAG00000030164*	RPL38	-0.79
BL_OCA_F000394	EE755870	ENSBTAG00000014804	HNRPDL	-0.81
A_70_P055581	EE784477	ENSBTAG00000001142	GNE	-0.82
BL_OCA_X000696	EE853694	ENSBTAG00000016420	CTNNB1	-0.82
A_70_P018376	DY478124	ENSBTAG00000008140	FAP	-0.83
BL_OCA_F000362	DY492542	ENSBTAG00000008140	FAP	-0.83
A_70_P059986	EE782950	ENSBTAG00000002006	THBS1	-0.85
A_70_P061311	EE872562	ENSBTAG00000010818	AEBP2	-0.87
BL_OTH_F000664	DY483719	ENSBTAG00000009778	NRAS	-0.87
A_70_P010686	EE755709	ENSBTAG00000010529	FZD6	-0.87
A_70_P062621	EE760476	-	-	-0.88
A_70_P065511	EE862970	-	-	-0.89
A_70_P029356	EE785047	ENSBTAG00000011613	PLS3	-0.89
A_70_P054711	EE770225	ENSBTAG00000017294	ORM1	-0.89
BL_OTH_F000609	DY479198	Multiple Targets		-0.89
BL_UN1_F000003	CD288332	-	-	-0.90
A_70_P019541	EE785539	ENSBTAG00000000184	EIF2AK3	-0.90
A_70_P053301	EE830605	ENSBTAG00000007718	TGIF1	-0.91
A_70_P070886	EE812808	ENSBTAG00000016045	TRIB2	-0.92
A_70_P039196	EE795086	-	-	-0.92
A_70_P057416	EE792423	ENSBTAG00000038434	ATRX	-0.93
BL_UN3_R000075	CU655351	ENSBTAG00000001391	ANKRD42	-0.93
BL_OCA_R000066	CU652331	ENSBTAG00000026851	FMNL2	-0.95
A_70_P001016	FE027322	ENSBTAG00000005969	EIF2S2	-0.97
BL_OCA_X000760	EE767289	ENSBTAG00000021965	SUB1	-0.97
A_70_P017906	EE836165	ENSBTAG00000002317	PTN	-0.97
A_70_P050511	EE784082	ENSBTAG00000007244	NID1	-0.98

BL_OTH_F000056	AF233078	ENSBTAG00000020935	HIF1A	-1.00
A_70_P053466	EE785467	ENSBTAG00000001851	CES1	-1.00
A_70_P060796	DY475743	ENSBTAG00000017745	IL6ST	-1.04
A_70_P072071	FE036440	ENSBTAG00000013410	MARK3	-1.13
A_70_P054061	EE831688	ENSBTAG00000010166	MICB	-1.19
BL_OTH_F000234	CD286927	ENSBTAG00000018271	SLC38A10	-1.22
A_70_P032741	CF116713	ENSBTAG00000038218	DEFB	-1.31
A_70_P041321	AF024645	ENSG00000211890	IGHA2	-1.58
BL_OTH_F000260	CD287559	ENSBTAG00000002069	BOLA	-2.09

* = probe complementary to reverse strand hit

0-140C vs 60-140T (120 differential probes):

	Agilent Probe Name	Target EST Accession	Ensembl Gene ID	Gene Symbol	Log Fold Change
**Up-regulated genes**	A_70_P031731	FE021050	-	-	1.04
	A_70_P001791	EE768843, NR_036644.1	-	RN28S1	1.01
	A_70_P019451	EE788143	ENSBTAG00000023411	LOC525947	0.92
	A_70_P070391	EE818692	ENSBTAG00000017021	MCM4	0.89
	A_70_P040451	EE828873	ENSBTAG00000004899	ABLIM1	0.83
	A_70_P043666	EE794724	ENSBTAG00000009374	PARP2	0.82
	A_70_P015131	EE828323	ENSBTAG00000012030	TTLL1	0.78
	A_70_P056176	EE869234	ENSBTAG00000001322	FAM154A	0.77
	A_70_P055321	DY491905	ENSBTAG00000010170	MBTPS1	0.77
	A_70_P017481	EE770023	ENSBTAG00000013526	EFTUD2	0.73
	A_70_P016641	NM_001078128, DY499458	-	SOX4	0.72
	A_70_P021286	EE751423	ENSBTAG00000021880	CABC1	0.72
	A_70_P047651	EE819067	ENSBTAG00000008758	KIF20A	0.70
**Down-regulated genes**	A_70_P060641	EE753991	ENSBTAG00000011082	IGF1	-0.70
	BL_OCA_F000516	CD286499	ENSBTAG00000020060	TXNIP	-0.70

A_70_P072016	FE036025	ENSBTAG00000000146	FARP1	-0.70
BL_OTH_F001418	U03092	Multiple Targets		-0.70
A_70_P047171	EE853535	ENSBTAG00000020420	AP1S2	-0.71
A_70_P066866	EE874417	ENSBTAG00000016229	KLF9	-0.71
A_70_P060651	EE849613	ENSBTAG00000024888	PDE5A	-0.71
BL_UN4_F000130	EE782267	-	-	-0.71
BL_OCA_X000465	EE772496	ENSBTAG00000014024	POMP	-0.71
BL_UN4_R000131	EE786340	ENSBTAG00000016486	SORBS2	-0.72
A_70_P034416	FE023178	ENSBTAG00000012411	HAPLN1	-0.72
A_70_P040476	EE795125	ENSBTAG00000038495	DCAMKL1	-0.72
A_70_P045201	EE789555	ENSBTAG00000024700	CYP2C19	-0.72
BL_CTL_F000097	U30299	ENSBTAG00000007159	ESR1	-0.73
BL_OTH_F001373	NM_001082596	ENSBTAG00000015356	TAC1	-0.73
BL_UN3_R000077	CU655415	-	-	-0.73
A_70_P046361	EE771637	ENSBTAG00000022699	CAV3	-0.73
BL_OCA_R000064	CU652314	-	-	-0.73
A_70_P032316	EE843892	ENSBTAG00000031707	FRMD6	-0.74
A_70_P001046	FE027431	-	-	-0.74
A_70_P039641	NM_001078657, DQ886530	ENSBTAG00000017704	ABCG2	-0.74
BL_OCA_F000774	DY494205	ENSBTAG00000014764	CD9	-0.74
BL_OTH_R000064	CN823747	ENSBTAG00000021211*	DPT	-0.74
A_70_P053466	EE785467	ENSBTAG00000001851	CES1	-0.75
BL_UN4_R000152	CU651873	ENSBTAG00000020338	CCT6B	-0.75
A_70_P038771	EE782257	ENSG00000112773	FAM46A	-0.75
BL_OCA_X000260	AF395335	ENSBTAG00000019866	NRP1	-0.76
A_70_P024051	EE834617, FE031091	ENSBTAG00000005726	HNRNPA2B1	-0.76
A_70_P044826	FE029955	-	-	-0.76
A_70_P069221	EE771985	ENSBTAG00000044017	MSRB3	-0.76
A_70_P040531	EE841180	ENSBTAG00000001862	PPP2R2B	-0.76

A_70_P043556	EE771614	-	-	-0.76
A_70_P030701	EE798501	ENSBTAG00000015794	NES	-0.76
A_70_P065851	EE756749	ENSBTAG00000009717	FGL2	-0.76
BL_OTH_R000235	EE758818	ENSBTAG00000000460	SYTL2	-0.76
BL_OCA_F000341	DY481807	ENSBTAG00000017704	ABCG2	-0.77
BL_OTH_F000425	CF118005	ENSBTAG00000027020	COL5A2	-0.77
BL_UN3_F000051	CU653839	ENSBTAG00000013136	EFCAB11	-0.77
A_70_P011801	FE030747	-	-	-0.78
BL_OTH_F000218	CD286513	ENSBTAG00000014614	ACTA2	-0.78
BL_OTH_R000299	EE772675	ENSBTAG00000001443*	CYP21A2	-0.79
A_70_P021241	DY482518	-	-	-0.80
A_70_P067996	DQ336700	ENSBTAG00000000128	FGF18	-0.80
A_70_P062066	EE864468	ENSBTAG00000012036	HR	-0.80
A_70_P016731	CN823173	ENSBTAG00000022890	MBP	-0.80
A_70_P020946	EE835047	ENSBTAG00000037811	CCL2	-0.80
BL_OCA_X000521	CU654365	ENSBTAG00000012545	CASC4	-0.81
A_70_P065561	EE753822	ENSBTAG00000007089	FRG1	-0.81
A_70_P066736	EE775565	ENSBTAG00000015204	SMPX	-0.81
BL_OTH_F000431	CF118159	ENSBTAG00000012994	LOX	-0.82
A_70_P016916	AF228667	ENSBTAG00000012855	LPL	-0.82
BL_UN4_F000069	CU653154	ENSBTAG00000000545	SMEK2	-0.82
BL_OTH_R000230	EE758534	ENSBTAG00000000442*	RBP4	-0.82
A_70_P060871	EE752485	ENSBTAG00000019587	PI15	-0.83
BL_OCA_F000886	S80867	ENSBTAG00000014567	MYLK	-0.84
A_70_P065771	EE758227	ENSBTAG00000010531	CYP 1B1	-0.84
A_70_P027206	EE767632	ENSBTAG00000010909	SLC30A1	-0.84
A_70_P000531	EE753209	-	-	-0.85
BL_OTH_F000630	DY480883	ENSBTAG00000011328	CGREF1	-0.86

BL_OTH_R000040	CF116541	ENSBTAG00000007900	FIS1	-0.86
A_70_P055351	DY491962	ENSBTAG00000017866	CD36	-0.86
A_70_P066546	FE035791	-	-	-0.86
A_70_P044621	FE023305	ENSBTAG00000024091	MALL	-0.87
BL_OTH_F000559	DY476231	ENSBTAG00000005353	DES	-0.87
A_70_P006546	EE865965	-	-	-0.87
A_70_P058711	DY491484	ENSBTAG00000017863	SRGN	-0.88
A_70_P059261	EE867186	ENSBTAG00000021176	CRISPLD2	-0.88
BL_OCA_R000256	EE776342	ENSBTAG00000006416	TMEM85	-0.88
A_70_P062621	EE760476	-	-	-0.88
BL_OCA_X000687	DY510754	ENSBTAG00000007988	STX2	-0.89
A_70_P032696	EE864067	ENSBTAG00000000078	GLIPR2	-0.89
A_70_P032741	CF116713	ENSBTAG00000038218	DEFB	-0.90
A_70_P022381	EE863953	ENSBTAG00000015082	FKBP5	-0.90
BL_OCA_F000025	CD287139	ENSBTAG00000002457	SEC61B	-0.91
A_70_P065511	EE862970	-	-	-0.91
BL_OTH_R000590	EE776342	ENSBTAG00000006416	TMEM85	-0,.92
BL_OCA_F000043	CF116963	ENSBTAG00000038495	DCLK1	-0.92
BL_OTH_F000475	CN823734	ENSBTAG00000009863	BHLHE40	-0.93
BL_OTH_R000192	DY519453	ENSBTAG00000021951*	RBM17	-0.93
A_70_P018256	DY493431	ENSBTAG00000019203	S100A4	-0.94
A_70_P063336	EE783131	ENSBTAG00000007596	GEM	-0.95
A_70_P042076	EE750097	ENSBTAG00000025398	UBD	-0.95
A_70_P057331	EE827130	ENSBTAG00000003708	SEC23A	-0.95
BL_OTH_F001344	NM_001009804	ENSBTAG00000010565	CYM	-0.99
BL_OTH_F001032	EE786654	ENSBTAG00000009876	C4BPA	-1.00
BL_OTH_F000325	CD289313	ENSBTAG00000015441	ACTG2	-1.00
BL_OTH_F001400	EE784427	Multiple Targets		-1.01
A_70_P008296	CN821983	ENSG00000185222	WBP5	-1.01

A_70_P062061	EE805740	-	-	-1.03
BL_OTH_F000497	DQ022555	ENSBTAG00000001755	NEUROD1	-1.08
A_70_P064081	EE858717	ENSBTAG00000017969	CA4	-1.09
A_70_P026671	EE748059, EE746827, EE750802	ENSG00000211895	IGHA1	-1.10
A_70_P031966	EE757951	ENSBTAG00000021906	COL29A1	-1.11
A_70_P017591	EE753919	ENSBTAG00000009876	C4BPA	-1.14
BL_OTH_F000727	DY490965	ENSBTAG00000009812	CXCL5	-1.14
BL_UN4_R000008	CU638020	-	-	-1.19
A_70_P012541	NM_001009456, AY327118	ENSBTAG00000011207	CNN1	-1.21
BL_OTH_R000503	FE030901	ENSBTAG00000021141	CD8A	-1.22
A_70_P053486	FE031722	-	-	-1.29
A_70_P031786	EE755865	ENSBTAG00000002069	BOLA	-1.39
A_70_P005526	CO202686	ENSBTAG00000020099	STMN2	-1.41
A_70_P054061	EE831688	ENSBTAG00000010166	MICB	-1.44
A_70_P062081	EE778788	ENSBTAG00000021615	LGALS2	-1.53
A_70_P041321	AF024645	ENSG00000211890	IGHA2	-1.60
A_70_P024826	EE866305	ENSBTAG00000034885	LOC614348	-1.61
A_70_P035986	DY505634	ENSBTAG00000015366	SFRP4	-1.63
A_70_P037831	EE770918	ENSBTAG00000012208	BT.62645	-1.67

* = probe complementary to reverse strand hit

(B) Differentially expressed fetal ovarian genes altered in two or more periods of exposure.

	Agilent Probe Name	Target EST Accession	Sigenae Contig Accession	Ensembl Gene ID	Gene Symbol	Log Fold Change			
						0-140T	0-80T	30-110T	60-140T
**Common to all conditions**	A_70_P041321	AF024645	AF024645.p.ov.7	ENSG00000211890	IGHA2	-2.13	-2.03	-1.58	-1.60
**Common 0-140T and 30-110T and 60-140T**	A_70_P008296	CN821983	CN821983.p.ov.7	ENSG00000185222	WBP5	-1.19	-	-0.72	-1.01

**Common 0-140T and 0-80T and 30-110T**	BL_OTH_F000135	AJ874675	AJ874675.p.ov.7	Multiple Targets		1,22	1,17	0,95	-
**Common 0-140T and 0-80T and 60-140T**	A_70_P026671	EE748059, EE746827, EE750802	EE748059.p.ov.7	ENSG00000211895	IGHA1	-1.46	-1.48	-	-1.10
**Common 0-140T and 30-110T**	A_70_P010686	EE755709	EE755452.p.ov.7	ENSBTAG00000010529	FZD6	-0.78	-	-0.87	-
**Common 0-140T and 60-140T**	A_70_P001791	EE768843, NR_036644.1	CF118064.p.ov.7	-	RN28S1	1.84	-	-	1.01
	A_70_P070391	EE818692	DY508444.p.ov.7	ENSBTAG00000017021	MCM4	0.96	-	-	0.89
	A_70_P040451	EE828873	EE794958.p.ov.7	ENSBTAG00000004899	ABLIM	0.82	-	-	0.83
	A_70_P021286	EE751423	DY482076.p.ov.7	ENSBTAG00000021880	CABC1	0.76	-	-	0.72
	BL_OCA_F000886	S80867	S80867.p.ov.7	ENSBTAG00000014567	MYLK	-0.72	-	-	-0.84
	A_70_P066546	FE035791	EE852996.p.ov.7	-	-	-1.03	-	-	-0.86
	A_70_P011801	FE030747	FE030747.p.ov.7	-	-	-1.16	-	-	-0.78
	BL_OTH_R000040	CF116541	CF116541.p.ov.7	ENSBTAG00000007900	FIS1	-1.16	-	-	-0.86
**Common 30-110T and 60-140T**	A_70_P031731	FE021050	FE021050.p.ov.7	-	-	-	-	1.68	1.04
	A_70_P043666	EE794724	EE794724.p.ov.7	ENSBTAG00000009374	PARP2	-	-	1.02	0.82
	A_70_P072016	FE036025	FE036024.p.ov.7	ENSBTAG00000000146	FARP1	-	-	-0.72	-0.70
	A_70_P065771	EE758227	DY475709.p.ov.7	ENSBTAG00000010531	CYP1B1	-	-	-0.75	-0.84
	A_70_P057331	EE827130	EE827130.p.ov.7	ENSBTAG00000003708	SEC23A	-	-	-0.77	-0.95
	A_70_P062621	EE760476	EE760476.p.ov.7	-	miR-450	-	-	-0.88	-0.88
	A_70_P065511	EE862970	EE753673.p.ov.7	ENSBTAG00000008114	CD99	-	-	-0.89	-0.91
	A_70_P053466	EE785467	EE751403.p.ov.7	ENSBTAG00000001851	CES1	-	-	-1.00	-0.75
	A_70_P054061	EE831688	DY513733.p.ov.7	ENSBTAG00000010166	MICB	-	-	-1.19	-1.44

Supplementary Table S3: Fetal ovarian epigenetic genes altered by sewage sludge exposure of pregnant ewes.[a]

Agilent Probe Name	Target EST Accession	Ensembl Gene ID	Gene Symbol	Log 2 (log Fold Change):				Function
				0-140	0-80	30-110	60-140	
A_70_P033491	EE750171	ENSBTAG00000009426	EZH2	0.39 **	0.26	0.23	0.33**	Histone-lysine N-methyltransferase
BL_OCA_X000527	EE774236	ENSBTAG00000009500	AOF2	0.38*	0.1	0.31**	0.25*	Lysine-specific Histone demethylase 1
BL_OTH_R000389	EE847497	ENSBTAG00000000222	ARID4B	0.14	0.02	0.44*	-0,17	Subunit of the histone deacetylase
A_70_P042586	EE827530	ENSBTAG00000005676	EHMT2	-0.11	-0.06	-0.32**	0.26 *	Histone-lysine N-methyltransferase
BL_OCA_R000170	CU655258	ENSBTAG00000006175	KDM5B	0.32	0.22	0.22	0.51**	Histone lysine demethylase 5b
A_70_P002341	EE748251	ENSBTAG00000018093	MLL	0.50	0.36	-0.54 *	0.24	Histone-lysine N-methyltransferase
BL_OCA_R000081	CU652728	ENSBTAG00000024199	MLL3	0.57 **	0.12	0.17	0.44**	Histone-lysine N-methyltransferase
A_70_P004941	EE809202	ENSBTAG00000009638	PRMT7	0.35	0.13	0.36	0.52**	Protein arginine methyltransferase 7
A_70_P022221	EE831325, EE828660	ENSBTAG00000009638	PRMT7	0.41**	0.11	-0.07	0.29	Protein arginine methyltransferase 7
A_70_P007511	EE803673	ENSBTAG00000000098	SETDB1	0.38**	0.26	0.32**	0.39 **	Histone-lysine N-methyltransferase
BL_UN1_F000025	CU655032	ENSBTAG00000007329	SETDB2	-0.15	0.02	0.03	-0.31**	Histone-lysine N-methyltransferase
A_70_P067526	EE805493	ENSBTAG00000014087	SUV420H2	0.23	0.09	0.03	0.57**	Histone-lysine N-methyltransferase
BL_OCA_X000025	EE811730	ENSBTAG00000003740	UTX	-0.30*	0	-0.35**	-0.18	H3K27 demethylase
A_70_P003906	FE021066	ENSBTAG00000001529	WHSC1L1	0.02	0.1	-0.11	0.33*	Histone-lysine N-methyltransferase

[a]Ewes were exposed throughout gestation (0-140 days) or during different periods of gestation: early (0-80 days), mid (30-110 days) and late (60-140 days) gestation.** $p \leq 0.05$, * $p \leq 0.1$

Supplementary Table S4: Fetal ovarian differentially expressed genes detected by microarray with follow up analysis by real time PCR.

Gene	Microarray				Real time PCR		
	Group comparisons	LogFC	Fold change	P value	Group comparisons	Fold change	P value
Ig heavy α 2 (IGHA2)	0-140C vs 0-140T	-2.13	4.39 ↓	0.012486	0-140C vs 0-140T	8.08 ↓	0.006
Calreticulin (CALR)	0-140C vs 0-140T	0.72	1.64 ↑	0.010258	0-140C vs 0-140T	1 .0	NS
Prostaglandin R assoc. protein (PTGFRN)	0-140C vs 0-140T	0.72	1.65 ↑	0.032037	0-140C vs 0-140T	1.04 ↑	NS
MHC class I HLA-B (LOC616942) A_70_P068241	0-140C vs 0-140T	1.01	2.01 ↑	0.031971	0-140C vs 0-140T	1.46 ↓	NS
28s RNA (RN28S1) A_70_P001791	0-140C vs 0-140T	1.8359	3.57 ↑	0.006109	0-140C vs 0-140T	1.47 ↑	0.028
AGT A_70_P038456	0-140C vs 30-110T	1.6829	0.75 ↑	0.005004	0-140C vs 30-110T	3.2 ↑	0.0098
AGTR1	0-140C vs 0-140T	-0.73	1.66 ↓	0.017735	0-140C vs 0-140T	0.6 ↓	NS
IGF1R	0-140C vs 60-140T	0.524	1.44 ↑	0.035098	0-140C vs 60-140T	1.7 ↑	0.0185
STAT3	0-140C vs 60-140 T	-0.616	1.53 ↓	0.015966	0-140C vs 60-140T	1.1	NS

Supplementary Table S5. Differentially expressed fetal ovarian proteins identified in one or more periods of exposure to chemicals in sewage sludge fertilizer.

Spot #	Protein Name	Gene Symbol	Mr (Da)	pI	MOWSE score	NCBI Accession Number (UniProt)	Sequence Coverage (%)	Significantly different spot volume vs 0-140C: (+) = increased, (-) = decreased	Network[n]
1326[s]	Aldo-keto reductase family 1, member B1 (aldose reductase)	*AKR1B1*	36296	5.76	163	P16116	13	0-80T (+)<br>30-110T (+)	1
1160	Serum Albumin	*ALB*	71186	5.88	908	AAI02743	43	60-140T (+)	1
1326[p]	Biliverdin reductase A	*BLVRA*	33907	5.85	497	NP_001091040	33	0-80T (+)<br>30-110T (+)	1
1346[s]	Biliverdin reductase A	*BLVRA*	33907	5.85	244	NP_001091040	19	0-80T (-)	1
750	Gelsolin	*GSN*	80966	5.54	120	NP_001029799 (Q3SX14)	6	60-140T (-)	1
1544[p]	Glutathione S-transferase Mu 1	*GSTM1*	25789	6.90	280	NP_787019	26	60-140T (+)	1
1544[s]	Glutathione S-transferase mu 3 (Brain)	*GSTM3*	27174	6.83	233	NP_001040025	23	60-140T (+)	1

1443[p]	High mobility group protein 1	*HMGB1*	25019	5.75	222	AAA57042	9	0-80T (+)<br>30-110T (+)<br>60-140T (+)	1
942	Lamin – M/C	*LMNA*	79805	7.70	434	CAA27173 (P02545)	14	0-140T (+)<br>0-80T (+)<br>30-110T (+)<br>60-140T (+)	1
660	Lamin-A/C	*LMNA*	79805	7.70	396	CAA27173	14	30-110T (+)	1
945	Lamin – A/C	*LMNA*	79805	7.70	335	CAA27173	12	0-140T (+)<br>0-80T (+)<br>30-110T (+)<br>60-140T (+)	1
1022	Protein disulfide-isomerase A3	*PDIA3*	57379	6.23	1280	NP_001156517	46	0-80T (+)<br>30-110T (+)<br>60-140T (+)	1
1044[s2]	Aldehyde (retinal) dehydrogenase 1 family, member A2	*ALDH1A2*	57158	5.79	112	BAA34785	10	30-110T (+)<br>60-140T (+)	1
1630	Transgelin	*TAGLN*	22609	8.87	625	NP_001103604	64	30-110T (+)	1
827	Serotransferrin	*TF*	79870	6.75	767	Q29443	24	30-110T (-)<br>60-140T (-)	1

966	Protein-glutamine gamma-glutamyltransferase 2	*TGM2*	78261	5.14	71	NP_803473	6	(+)0-80T (+)<br>30-110T (+)	1
1044[s1]	D-3-phosphoglycerate dehydrogenase	*PHGDH*	57327	6.47	345	NP_001030189	10	30-110T (+)<br>60-140T (+)	2
1767	Hemoglobin fetal subunit beta	*HBB*	15978	6.59	1033	P02083	94	60-140T (-)	2
1443[s]	Haloacid-dehalogenase-like hydrolase domain-containing protein 2	*HDHD2*	26749	5.81	72	BAB22395<br>Q3UGR5	12	0-80T (+)<br>30-110T (+)<br>60-140T (+)	2
1201[p]	COP9 signalosome complex subunit 4	*COPS4*	43454	5.57	335	AAD43021	29	30-110T (+)<br>60-140T (+)	2
1201[s]	Selenophosphate synthetase 1	*SEPHS1*	43396	5.64	134	NP_036379	10	30-110T (+)<br>60-140T (+)	2
1346[p]	Serine/threonine protein phosphatise	*PPP1CB*	37962	5.84	559	BAC40733	40	0-80T (-)	2
970[p]	Acetyl-CoA synthetase family member 2, mitochondrial	*ACSF2*	68955	7.88	158	NP_001071580	6	60-140T (+)	2
970[s]	Paraspeckle component 1	*PSPC1*	41884	5.93	60	BAA91924	5	60-140T (+)	2

1044[p]	Dihydropyrimidinase-related protein 2	*DPYSL2*	62638	5.95	618	NP_001069468	26	30-110T (+)<br>60-140T (+)	2
544	Collagen alpha-2(VI) chain	*COL6A2*	109736	5.85	149	AAH65509	4	30-110T (+)	2
1263	Short/branched chain specific acyl-CoA dehydrogenase, mitochondrial	*ACADSB*	47797	6.53	91	NP_001600	5	0-80T (-)<br>60-140 (+)	2
1524	Protein gene product 9.5	*UCHL1*	20319	5.02	147	AAP07110 (Q80XX5)	22	0-80T (+)<br>60-140T (+)	2
886	Stress-70 protein, mitochondrial	*HSPA9*	74019	5.97	330	AAA67526	12	60-140T (+)	2
664	Methylenetetrahydrofolate dehydrogenase (NADP+ dependent) 1	*MTHFD1*	101740	6.91	273	NP_001076946 (A4FUD0)	8	30-110T (+)<br>60-140T(+)	2

Expression is relative to controls (0-140C). The accession number is derived from NCBI or UniProt.

* next to the spot number depicts spots containing significant protein matches that cannot be discriminated

[p] Primary identification

[s] Secondary identification in same protein spot

[n]IPA network analysis identified two networks: Network 1, Free Radical Scavenging, Cancer, Organismal Injury and Abnormalities, score = 31, focus molecules = 13, other molecules in network: Ap1, caspase, Creb, ERK, ERK1/2, Focal adhesion kinase, Growth hormone, Histone h3, Histone h4, IgG, IL1, IL12 (complex), Jnk, NFkB (complex), P38 MAPK, p85 (pik3r), PDGF BB, PI3K (complex), Pkc(s), PLC, Tgf beta, Vegf; Network 2, Hereditary Disorder, Metabolic Disease, Amino Acid Metabolism, score =35, focus molecules = 142, other molecules in network: ACAD10, Akt, CEP89, COL6A5, DNAJB3, DNAJC16, DNAJC17, DNAJC22, DNAJC28, GRPEL2, Hba1/Hba2, Mapk, MTHFD2L, NUDT6, SLC22A17, SPRED3, UBC, USP35, USP40, USP27X, YIPF1.

Supplementary Table S6: Top molecular and cellular functions affected by sewage sludge exposure.

Name	p-value	#Molecules
Free Radical Scavenging	3.94E-03 - 4.91E-08	9
Cell-To-Cell Signaling and Interaction	1.44E-02 - 7.90E-06	10
Small Molecule Biochemistry	1.44E-02 - 9.99E-06	16
Drug Metabolism	1.44E-02 - 3.38E-05	8
Protein Synthesis	6.56E-03 - 3.38E-05	8

Supplementary Table S7. Predicted downstream effects of the transcripts and proteins altered in the day 60-140 exposure group compared with controls (Supplementary table 2 and Supplementary table 5). The Activation z-score predicts the activation state of the upstream regulator, using the gene expression patterns of the genes downstream of an upstream regulator. An absolute z-score of ≥ 2 is considered significant.

Diseases or Functions	Predicted Activation State	Activation z-score	Molecules	# Molecules
Migration of cells	Decreased	-3.158	ACTA2, ALB, BHLHE40, CCL2, CD36, CD8A, CD9, CES1, CXCL5, CYP1B1, CYP21A2, DCLK1, DPT, DPYSL2, ESR1, FRMD6, GLIPR2, GSN, HMGB1, HNRNPA2B1, IGF1, LMNA, LOX, MYLK, NES, NEUROD1, NRP1, S100A4, SFRP4, TAC1	30
Cell movement	Decreased	-3.101	ACTA2, ALB, BHLHE40, CAV3, CCL2, CD36, CD8A, CD9, CES1, CNN1, CXCL5, CYP1B1, CYP21A2, DCLK1, DPT, DPYSL2, ESR1, FGL2, FRMD6, GLIPR2, GSN, HMGB1, HNRNPA2B1, IGF1, LMNA, LOX, MBP, MYLK, NES, NEUROD1, NRP1, PDIA3, S100A4, SFRP4, TAC1	35
Microtubule dynamics	Decreased	-2.850	ABLIM1, CAV3, CCL2, CD9, DCLK1, DPYSL2, ESR1, FARP1, GEM, GSN, GSTM1, HMGB1, IGF1, KLF9, LOX, MBP, NEUROD1, NRP1, PDIA3, PHGDH, S100A4, STMN2, STX2, TAC1, TTLL1, UCHL1	26
Organization of cytoplasm	Decreased	-2.824	ABLIM1, CAV3, CCL2, CD9, DCLK1, DES, DPYSL2, ESR1, FARP1, FIS1, GEM, GSN, GSTM1, HMGB1, IGF1, KLF9, LOX, MBP, MBTPS1, NEUROD1, NRP1, PDIA3, PHGDH, S100A4, STMN2, STX2, TAC1, TF, TTLL1, UCHL1	30
Organization of cytoskeleton	Decreased	-2.824	ABLIM1, CAV3, CCL2, CD9, DCLK1, DES, DPYSL2, ESR1, FARP1, GEM, GSN, GSTM1, HMGB1, IGF1, KLF9, LOX, MBP, NEUROD1, NRP1, PDIA3, PHGDH, S100A4, STMN2, STX2, TAC1, TF, TTLL1, UCHL1	28
Development of cardiovascular system	Decreased	-2.543	ALDH1A2, AP1S2, CCL2, CD36, CD9, CXCL5, CYP1B1, ESR1, FGF18, GEM, GSN, GSTM1, HMGB1, IGF1, LOX, MTHFD1, NRP1, RBP4, S100A4, SOX4, SRGN, TAC1, TF	23
Angiogenesis	Decreased	-2.543	ALDH1A2, AP1S2, CCL2, CD36, CD9, CXCL5, CYP1B1, ESR1, FGF18, GEM, GSN, GSTM1, HMGB1, IGF1, LOX, NRP1, S100A4, SRGN, TAC1, TF	20
Formation of cellular protrusions	Decreased	-2.495	ABLIM1, CCL2, CD9, DCLK1, DPYSL2, FARP1, GEM, GSN, HMGB1, IGF1, KLF9, LOX, MBP, NEUROD1, NRP1, PDIA3, PHGDH, S100A4, STX2, TAC1, TTLL1, UCHL1	22

Invasion of tumor cell lines	Decreased	-2.479	ABLIM1, ACTA2, CCL2, CD9, CXCL5, CYP1B1, DPYSL2, ESR1, HMGB1, HNRNPA2B1, IGF1, LOX, NES, NRP1, S100A4, SOX4, UBD	17
Advanced malignant tumor	Decreased	-2.439	ABLIM1, ACTA2, ALDH1A2, CCL2, CD36, CD9, CNN1, CXCL5, CYP1B1, DPYSL2, ESR1, FGF18, GSTM3, HMGB1, IGF1, KIF20A, LGALS2, LOX, NES, NEUROD1, NRP1, PARP2, S100A4, STMN2, STX2, TXNIP, UBD	27
Metastasis	Decreased	-2.439	ABLIM1, ACTA2, CCL2, CD36, CD9, CNN1, CXCL5, CYP1B1, DPYSL2, ESR1, GSTM3, HMGB1, IGF1, KIF20A, LGALS2, LOX, NES, NEUROD1, NRP1, PARP2, S100A4, STMN2, STX2, TXNIP, UBD	25
Migration of tumor cell lines	Decreased	-2.438	ACTA2, CCL2, CD36, CD9, CXCL5, DPYSL2, ESR1, HNRNPA2B1, IGF1, LOX, NES, NRP1, S100A4, SFRP4, TAC1	15
Vasculogenesis	Decreased	-2.417	ALDH1A2, AP1S2, CCL2, CD36, CD9, CYP1B1, ESR1, GEM, GSN, GSTM1, HMGB1, IGF1, LOX, NRP1, S100A4, TAC1, TF	17
Production of reactive oxygen species	Decreased	-2.306	CD36, GSN, HBB, HMGB1, HSPA9, IGF1, MYLK, TAC1, TXNIP	9
Cell movement of tumor cell lines	Decreased	-2.260	ACTA2, CCL2, CD36, CD9, CNN1, CXCL5, DPYSL2, ESR1, HNRNPA2B1, IGF1, LOX, NES, NEUROD1, NRP1, S100A4, SFRP4, TAC1	17
Cell viability of tumor cell lines	Decreased	-2.219	ABCG2, ALB, BHLHE40, CCL2, FKBP5, GSTM1, HBB, HMGB1, IGF1, NEUROD1, NRP1, POMP, PPP2R2B, S100A4	14
Sprouting	Decreased	-2.183	CCL2, DPYSL2, FARP1, GEM, HMGB1, IGF1, KLF9, NEUROD1, NRP1, PDIA3	10
Cell viability	Decreased	-2.151	ABCG2, ALB, BHLHE40, CCL2, CD8A, CD9, ESR1, FGF18, FKBP5, GSTM1, HBB, HMGB1, IGF1, LMNA, MBP, NEUROD1, NRP1, PDIA3, POMP, PPP2R2B, S100A4, TXNIP, UCHL1	23
Branching of cells	Decreased	-2.143	CCL2, DPYSL2, FARP1, GEM, HMGB1, IGF1, KLF9, NEUROD1, NRP1, PDIA3, STX2	11
Quantity of metal	Decreased	-2.111	CCL2, CD8A, ESR1, GSN, IGF1, S100A4, SLC30A1, TAC1, TF, TXNIP	10
Stimulation of cells	Decreased	-2.048	CCL2, CD8A, CXCL5, HMGB1, IGF1, MBP, NEUROD1, S100A4, TAC1	9
Metastasis of	Decreased	-2.023	ABLIM1, CCL2, CNN1, CXCL5, IGF1, LOX, NEUROD1, S100A4, STX2,	11

cells			TXNIP, UBD	
Formation of plasma membrane projections	Decreased	-2.014	DCLK1, DPYSL2, FARP1, GEM, HMGB1, IGF1, KLF9, LOX, MBP, NEUROD1, NRP1, PDIA3, PHGDH, TAC1, UCHL1	15
Concentration of cholesterol	Increased	2.141	CAV3, CD36, CES1, ESR1, LPL, MBTPS1, TXNIP	7
Concentration of Triacylglycerol	Increased	2.372	BHLHE40, CAV3, CD36, CES1, IGF1, LMNA, LPL, TXNIP	8

Supplementary Table S8: Quantitative PCR Probes and Primer sequences.

Target	Primer sequence	GenBank accession number
GAPDH	F: GGTTCACGCCCATCACA	NM_001190390
	R: ACTACCATGGAGAAGGCTGG	
	PR: AGAGGGTCATCATCTCTGCACCTTCT	
HPRT	F: GAACGGCTGGCTCGAG	EE751310
	R: CCAACAGGTCGGCAAAG	
	PR: AATGTGATGGCCACCCATCTCCT	
YWHAZ	F: GGAGCCCGTAGGTCATCTTG	AY970970
	R: CTCGAGCCATCTGCTGTTTTT	
	PR: CAGCACCTTCCGTCTTTTGCTCAATA CTGGAGA	
AGTR1	F: CCAATTTCCAAAGGGCAGCAA	AJ874684
	R: GCTTAGCGAGTCTTTGTATGAAATGT	
	PR: CTGTGGCCCACGTGTACCTGCTACTG	
IGF1R	F: CAACCCAGGGAACTACACAG	NM_001040479.1
	R: ATATGTCGTCTTGGCCTGAA	
	PR: CTCTCTCTGGGAATGGGTCATGGAC	
AGT	F: TCCCACGCTCACTAGACTTG	AF035417.1
	R: TTTCCTTGGAAGTGGACGTA	
	PR: CCAAATCTCGCTGCTGAGAAGATCA	
STAT3	F: GCTGGAGCAGAAATCGTAAA	AF257464.1
	R: CTTTAAGGGAGGGTCTCAGC	
	PR: TCCGTGGCTCTTAGGTCATGTGAG	
IGHA2	F: AACCCACCCACGTGAACGT	AF024645
	R: GGCGAGCGTGGAGTTTATTC	
	PR: CTACTGAGTGCCCCCACCCCCA	
CALR	F: GCTGTTTCCAGCTGGTTTGG	CD288071

	R: CAGCACATTCTTGCCCTTGT	
	PR: CCAGACATCTGTGGACCCGGCAC	
PTGFRN	F: TGAGCGCAGACATGTTTACCA	CU653858
	R: ACACTCGATTGTTCCATCAGAAAG	
	PR: CACACGGCTCTTCTTCCCACGGC	
MHC CLASS I (HLA-B)	F: AGGAGACGCAGGGAACTAAGG	AJ874684
	R: GTAGCCGCGCAGGTTGT	
	PR:CACTGCACTGACTTTCCGAGCGAACT	
RN28S1	F: TGGTTCCCTCCGAAGTTTCC	CF118064
	R: CTAATCATTCGCTTTACCGGATAAA	
	PR: CAGGATAGCTGGCGCTCTCGCAAC	
